# Bruton’s tyrosine kinase inhibition reduces disease severity in a model of secondary progressive autoimmune demyelination

**DOI:** 10.1186/s40478-023-01614-w

**Published:** 2023-07-12

**Authors:** Kirsten Scarlett Evonuk, Sen Wang, Josh Mattie, C. J. Cracchiolo, Reine Mager, Željko Ferenčić, Ethan Sprague, Brandon Carrier, Kai Schofield, Evelyn Martinez, Zachary Stewart, Tara Petrosino, Gregory Andrew Johnson, Isharat Yusuf, Warren Plaisted, Zachary Naiman, Timothy Delp, Laura Carter, Suzana Marušić

**Affiliations:** 1 Hooke Laboratories, LLC, 439 South Union Street, Lawrence, MA 01843 USA; 2 Gossamer Bio, 3013 Science Park Road, Suite 200, San Diego, CA 92121 USA

**Keywords:** Multiple sclerosis, Secondary progressive, Demyelination, Microglia, B cells, Myeloid cells, Experimental autoimmune encephalomyelitis

## Abstract

Bruton’s tyrosine kinase (BTK) is an emerging target in multiple sclerosis (MS). Alongside its role in B cell receptor signaling and B cell development, BTK regulates myeloid cell activation and inflammatory responses. Here we demonstrate efficacy of BTK inhibition in a model of secondary progressive autoimmune demyelination in Biozzi mice with experimental autoimmune encephalomyelitis (EAE). We show that late in the course of disease, EAE severity could not be reduced with a potent relapse inhibitor, FTY720 (fingolimod), indicating that disease was relapse-independent. During this same phase of disease, treatment with a BTK inhibitor reduced both EAE severity and demyelination compared to vehicle treatment. Compared to vehicle treatment, late therapeutic BTK inhibition resulted in fewer spinal cord-infiltrating myeloid cells, with lower expression of CD86, pro-IL-1β, CD206, and Iba1, and higher expression of Arg1, in both tissue-resident and infiltrating myeloid cells, suggesting a less inflammatory myeloid cell milieu. These changes were accompanied by decreased spinal cord axonal damage. We show similar efficacy with two small molecule inhibitors, including a novel, highly selective, central nervous system-penetrant BTK inhibitor, GB7208. These results suggest that through lymphoid and myeloid cell regulation, BTK inhibition reduced neurodegeneration and disease progression during secondary progressive EAE.

**Supplementary Information:**

The online version contains supplementary material available at 10.1186/s40478-023-01614-w.

## Introduction

Multiple sclerosis (MS) is an immune-mediated demyelinating disease of the central nervous system (CNS) affecting over 2.8 million people worldwide [57]. Patients most commonly present with relapsing–remitting MS (RRMS), characterized by periods of neurological disability (relapses) followed by periods of disability reduction (remission). Most patients with RRMS eventually develop secondary progressive MS (SPMS), during which disability accumulates more steadily over time. SPMS is further categorized as “active” when there is evidence of new relapses or new/enlarging lesions by magnetic resonance imaging, or “not active” when there is no evidence of disease activity [26]. Relapses can be almost completely suppressed by drugs that prevent new lymphocyte infiltration into the CNS, such as natalizumab and fingolimod [22, 23, 40]. Natalizumab inhibits CNS entry by blocking the alpha 4 subunit of integrins, preventing lymphocytes from crossing the blood–brain barrier [47], while fingolimod achieves a similar effect by modulating the sphingosine-1-phosphate receptor and preventing egress of lymphocytes from peripheral lymph nodes [7]. Because SPMS involves neurodegenerative processes, it fails to respond to these treatments, presenting a great unmet medical need [13]. A major impediment to the development of new drugs is a lack of robust animal models of SPMS. Identifying animal models suitable for testing molecular pathways that play a role in disease progression in SPMS will open new avenues of treatment for patients.

An important role for B cells in disease pathogenesis is suggested by the efficacy of B cell depletion with anti-CD20 antibodies in treating RRMS, active SPMS, and primary progressive MS (PPMS) [16, 36, 58]. Furthermore, approximately 40% of patients with SPMS have ectopic B cell follicles which are associated with more pronounced cortical demyelination and microglia activation, and worse disease prognosis [30]. Bruton’s tyrosine kinase (BTK) has emerged as a target of interest for B cell function modulation during MS. Unlike anti-CD20 therapy, BTK inhibition reportedly disrupts B cell maturation and signaling without causing widespread B cell depletion [52]. This is a potentially important benefit, as B cell depletion can induce humoral immunosuppression that increases susceptibility to severe COVID-19 [19], impair the humoral response to vaccination [49], and increase the risk of infection [39]. Additionally, BTK plays a role in the production of pro-inflammatory cytokines by monocytes [6], phagocytosis by macrophages [20] and microglia [24], and macrophage survival [33], indicating that BTK inhibition could protect the CNS via modulation of myeloid cell activation.

BTK inhibitors have demonstrated efficacy in phase 2 clinical trials for treatment of relapsing MS, which includes both RRMS and active SPMS [35, 45]. Several phase 3 clinical trials are underway testing the efficacy of BTK inhibitors in treating RRMS and active SPMS, non-active SPMS, and PPMS (RRMS and active SPMS: FENhance/NCT04586010 and NCT04586023, Evolution RMS 1 and 2/NCT04338022 and NCT04338061, GEMINI 1 and 2/NCT04410978 and NCT04410991, NCT05147220; non-active SPMS: HERCULES/NCT04411641; PPMS: PERSEUS/NCT04458051, FENtrepid/NCT04544449). Peer-reviewed research has yet to be published on the efficacy of BTK inhibition in an animal model of SPMS.

Experimental autoimmune encephalomyelitis (EAE) in Biozzi mice immunized with spinal cord homogenate (SCH)/complete Freund’s adjuvant (CFA) recapitulates the progression from RRMS to SPMS [2, 15, 37]. As in patients with progressive MS, a feature of this EAE model is progression independent of relapse activity (PIRA) [1, 43]. Additionally, meningeal tertiary lymphoid tissue in the spinal cord and high anti-myelin oligodendrocyte glycoprotein (MOG) antibody levels in serum are observed during late chronic disease [37], indicating B cell involvement in the EAE pathogenesis of this model. Here we demonstrate that in Biozzi mice with secondary progressive EAE (SPEAE), late therapeutic treatment with FTY720 (fingolimod), which blocks EAE relapses, is ineffective at reducing disease progression. This indicates that EAE progression is independent of influx of new lymphocytes, resembling human SPMS. This model is therefore uniquely suited to studying the effects of BTK inhibition in both relapsing–remitting EAE (RREAE) and SPEAE.

The first BTK inhibitor approved by the United States Food and Drug Administration (FDA) was ibrutinib, for the treatment of mantle cell lymphoma (in 2013). In the present study, we aimed to determine if treatment of Biozzi mice with ibrutinib during RREAE and SPEAE would ameliorate EAE symptoms. We found that ibrutinib treatment during both phases of EAE in Biozzi mice reduced disease severity. Treatment during SPEAE reduced the number of CD45^hi^CD11b^+^ spinal cord-infiltrating myeloid cells (IMCs) and altered expression of activation markers in IMCs and CD45^int^CD11b^+^ microglia. These changes were accompanied by reduced spinal cord pathology, including decreased tissue area of SMI32, a marker of axonal damage. Interestingly, B cell infiltration in the CNS was not significantly altered. We also demonstrate efficacy of novel, CNS-penetrant, highly selective BTK inhibitor GB7208, at a tenfold lower dose than ibrutinib, in treating SPEAE in Biozzi mice. Collectively, these results suggest that BTK inhibition induces a switch from an inflammatory to a homeostatic or anti-inflammatory environment, resulting in both reduced demyelination and reduced axonal damage during SPEAE.

## Materials and methods

### Mice

Biozzi mice were purchased from The Jackson Laboratory (strain 027904) and bred in-house at Hooke Laboratories. EAE was induced in 6- to 11-week-old (ages balanced across groups) female Biozzi mice at Hooke Laboratories.

Female SJL/J mice were purchased from The Jackson Laboratory (strain 000686). EAE was induced in 8-week-old female SJL/J mice at Hooke Laboratories.

Ten-week-old female C57BL/6J mice were purchased from The Jackson Laboratory (strain 000664) for target occupancy determination at Explora BioLabs.

Prior to all experiments, all mice were acclimated to their respective facilities for at least 7 days.

### EAE induction

EAE was induced in Biozzi mice by immunization with emulsion prepared by emulsifying 5, 7.5, or 15 mg/mL Swiss Webster mouse (model number SW-F, Taconic Biosciences) SCH with 0.7 mg/mL *M. tuberculosis* in complete Freund’s adjuvant (CFA) at a 1:1 ratio. This emulsion was injected subcutaneously at two sites in the lower back at 0.1 mL/site on Day 0. The booster emulsion was administered 7 days later and was prepared by emulsifying 7.5 or 15 mg/mL SCH with 0.35 or 0.7 mg/mL *M. tuberculosis* in CFA at a 1:1 ratio. This emulsion was injected subcutaneously at two sites in the upper back at 0.1 mL/site.

EAE was induced in SJL/J mice using Hooke Kit™ [Ser^140^]-PLP_139-151_/CFA Emulsion, (Hooke Laboratories, cat. no. EK-0120), following Hooke’s recommended protocol.

To evaluate EAE development, mice were weighed three times per week beginning one to zero days prior to EAE immunization, and scored daily from Day 7 post-immunization by experts blinded to treatment and previous scores using the following scale: 0, no obvious abnormalities; 1, limp tail; 2, limp tail and weakness of hind legs; 3, limp tail and complete paralysis of hind legs, or limp tail with paralysis of one front and one hind leg; 4, limp tail and complete hind leg and partial front leg paralysis (mice scoring 4 two days in a row were euthanized and assigned a score of 5 for the remainder of the experiment); 5, complete hind and complete front leg paralysis with no movement around the cage, or mouse found dead due to paralysis, or mouse euthanized due to severe paralysis. In-between scores were assigned when clinical signs fell between the previously defined scores.

### Drug formulation and dosing

Ibrutinib (MedChemExpress, cat. no. HY-10997) was prepared in a vehicle consisting of 0.5% methylcellulose and 0.2% Tween 80 in water and dosed orally, daily, at 50 mg/kg in a volume of 10 mL/kg. The dose was selected based on the Center for Drug Evaluation and Research Pharmacology Review for Imbruvica (ibrutinib), in which complete BTK occupancy by ibrutinib in splenocytes from BALB/c mice was found two hours after oral dosing at 50 mg/kg [56]. The present study also confirmed high brain BTK occupancy at a dose of at least 45 mg/kg ibrutinib in naïve C57BL/6J mice (Fig. 7c).

FTY720 (fingolimod; Selleck Chemicals, cat. no. S5002) was prepared in a vehicle consisting of 2% ethanol in water and dosed orally, daily, at 3 mg/kg in a volume of 5 mL/kg.

GB7208 (preclinical drug product candidate provided by Gossamer Bio) was prepared in a vehicle consisting of 0.5% methylcellulose and 0.1% Tween 80 in water and dosed orally, daily, at 5 mg/kg in a volume of 5 mL/kg. The dose used in the present study was determined based on brain target occupancy and brain GB7208 concentration in naïve C57BL/6J mice (Figs. 7c, d).

All treatments were administered at the same time ± 1 h each day.

### Histology, immunohistochemistry, and imaging

Mice were euthanized using CO_2_ asphyxiation and perfused with PBS. Spines were removed and immersed in 10% neutral buffered formalin for at least 72 h. After fixation, spines were decalcified, cut transversely into cervical, thoracic, and lumbar regions, and embedded in paraffin. Four-µm-thick transverse sections were prepared and stained with hematoxylin and eosin using a Tissue-Tek Prisma Plus Automated Slide Stainer (Sakura Finetek). For immunohistochemistry, spine sections were stained using a Ventana DISCOVERY ULTRA IHC/ISH research platform (Roche Diagnostics). To stain myelin, slides were incubated with rabbit anti-MBP (1:1000; Abcam, cat. no. ab40390) followed by biotinylated goat anti-rabbit (1:1000; ThermoFisher, cat. no. 65–6140), and antibodies visualized using the avidin–biotin-immunoperoxidase complex method with a streptavidin-conjugated 3,3'-diaminobenzidine horseradish peroxidase substrate. To stain myeloid cells, slides were incubated with rabbit anti-Iba1 (1:1000; FUJIFILM Wako Pure Chemical Corporation, cat. no. 013–26,791) followed by components of a Biotin Free OmniMap DAB anti-Rabbit HRP kit per the company’s instructions (Roche Diagnostics, cat. no. 05266548001). Slides stained by immunohistochemistry were dehydrated through graded alcohols, cleared in xylene, and coverslipped prior to imaging. All slides were digitized at 20X magnification using a Nanozoomer 2.0-HT slide scanner (Hamamatsu).

### Assessment of demyelination, inflammation, apoptosis, and proportion of tissue area stained

Demyelination was assessed in randomized and blinded anti-MBP-stained sections by an expert pathologist (ŽF). Demyelination scores were assigned depending on the proportion of spinal cord white matter area demyelinated using the following scale: 0, < 2% demyelinated area; 1, 2 to 5% demyelinated area; 2, 6 to 19% demyelinated area; 3, 20 to 29% demyelinated area; 4, 30 to 50% demyelinated area; 5, > 50% demyelinated area.

The numbers of inflammatory foci containing at least 20 cells, and apoptotic cells (apoptotic nuclei with karyorrhexis, see Fig. 3i, inset), were counted in hematoxylin- and eosin-stained, randomized, and blinded sections by an expert pathologist (ŽF). When foci were coalescing, the number of foci was estimated.

The proportion (%) of area positive for anti-Iba1 antibody stain was measured in whole spinal cord sections, and the proportion (%) of area positive for anti-SMI32 antibody stain was measured in spinal cord white matter, using ImageJ version 1.53p (National Institutes of Health). Spinal cord tissue and white matter were digitally isolated from each image and the area of each isolated spinal cord region (cervical, thoracic, and lumbar), or area of white matter within each spinal cord region, was measured. Within these regions, areas of a fixed minimum size and fixed DAB^+^ intensity were measured using identical thresholds across all images to eliminate analytical biases. Sections with extreme morphological damage were excluded from analysis (maximum 1 section per mouse). To enhance visibility, GNU Image Manipulation Program (GIMP) version 2.10 (The GIMP Development Team) was used to adjust contrast and brightness (and, where indicated in figure legends, gamma and input levels) of illustrative images evenly across figures.

### Flow cytometry

Mice were euthanized using CO_2_ asphyxiation and perfused with PBS. Spleen, spinal cord, and/or brain were isolated from either all mice or from representative mice from each group. Representative mice were selected to have the same average score, same disease course, and same variability as the whole group. Single-cell suspensions were prepared for each tissue and each mouse separately.

Spinal cord and brain cells were isolated using a 37%/70% Percoll gradient. Isolated cells were stained immediately, or for panels with cytokine staining, were stimulated with phorbol myristate acetate (50 ng/mL), ionomycin (1 µg/mL), and brefeldin (1 µg/mL) for 4 h at 37 °C in 5% CO_2_. For panels including IL-10, monensin (1.36 µg/mL) was also added.

Cells were incubated with Fc block and stained with antibodies for cell surface staining. For intracellular panels, cells were then fixed and permeabilized using a Cytofix/Cytoperm kit (BD Biosciences) and stained overnight with antibodies for intracellular staining. All antibodies used for flow cytometric analysis are listed in Additional file 1: Table S1. Dead cells were excluded by staining with Fixable Viability Dye eFluor 780 (eBioscience, cat. no. 65–0865-14). Stained cells were acquired on either an LSRII flow cytometer (BD Biosciences) or FACSymphony A1 cell analyzer (BD Biosciences) and analyzed with FlowJo version 10.8.1 (Treestar).

### Kinase selectivity panel

Kinome profiling was conducted by AssayQuant Technologies (Marlboro, MA) using Sox chromophore technology and Sox-based peptide substrates developed for human kinases in a 384-well format. Compounds were tested at 1 µM in the presence of 1 mM ATP. Single lots of GB7208 and ibrutinib were assayed and percent inhibition averaged for each kinase.

### Target occupancy determination

C57BL/6J mice were randomized into groups based on body weight. Mice were dosed orally, daily, for a total of 3 days with ibrutinib, GB7208, or their respective vehicles. One hour after the final dose, all animals were perfused with PBS using a syringe pump at a rate of 3 mL/minute under isoflurane anesthesia until fully exsanguinated (total time of 1 min), followed by cervical dislocation. Brains were collected, dissected into two hemispheres, and immediately snap-frozen using liquid nitrogen.

The concentration of ibrutinib or GB7208 in brain samples was determined by liquid chromatography with tandem mass spectrometry (LC–MS/MS).

For target occupancy analysis, lysis buffer was prepared from 10 × Cell Lysis Buffer (Cell Signaling Technology, cat. no. 9803S) and 100 × Halt Protease Inhibitor Cocktail (ThermoFisher Scientific, cat. no. 78429). Left brain hemispheres were thawed on wet ice, and 750 µL of ice-cold lysis buffer added to each sample, and samples plus lysis buffer transferred to Miltenyi M tubes. M tubes were loaded onto a gentleMACS Octo Dissociator (Miltenyi Biotec, cat. no. 130-096-427) and lysis achieved using program “Protein_01” followed by centrifugation to pellet debris. Approximately 0.75 mL of soluble lysate from each half-brain was transferred to a microcentrifuge tube and gently rotated overnight at 4 °C following mechanical tissue dissociation. Tubes were centrifuged the next day at 13,000 rpm at 4 °C and soluble lysates transferred into a new microcentrifuge tube.

The occupancy of BTK in brain lysates was tested by loading a V-bottom 96-well plate containing 1 µL of 0.02 mM BTK active site probe (provided by Gossamer Bio) with 1 µL of 0.02 mM of each sample, and shaking at room temperature for 1 h. The amount of unoccupied BTK was tested using an enzyme-linked immunosorbent assay (ELISA) with primary antibody α-BTK (clone D3H5, Cell Signaling Technology, cat. no. 8547S) diluted 1:500, detection antibody AffiniPure Donkey Anti-Rabbit IgG (Jackson Immuno Research, cat. no. 711–005-152) diluted 1:2500, and TMB substrate (ThermoFisher, cat. no. 34029). The plate was read in a Pherastar plate reader (BMG, Germany) at the wavelengths 450 nm and 570 nm (correction wavelength). The correction of OD450 nm—OD570 nm was done to obtain the optical density (OD) value for analysis of occupancy. The vehicle treated group was averaged. The ODs for all other animals were divided by their respective vehicle groups, and those values were then multiplied by 100 to obtain the percentage value of the vehicle-treated group.

### Statistics

Statistical analyses were performed and graphs generated using GraphPad Prism version 8.2.1 (GraphPad Software). Specific analyses performed are reported in figure and table legends. Graphs and images were arranged using Inkscape version 1.2.1 (https://inkscape.org). A p value less than 0.050 was considered significant. Underlying data for all tables and figures are provided in Additional file 2.

For mice that died from EAE, a clinical score of 5 was assigned for the remainder of the study and the last body weight was continued until the end of the study. For mice that died of a different or unknown cause, clinical scores and body weight recordings stopped on the day of death, scores were excluded from the mean maximum score and average end score, and percent relative end body weights and areas under the curves of relative body weight over time excluded from analysis. In calculation of relapses, mice that died were excluded from analysis unless they relapsed prior to death.

## Results

### Biozzi mice develop SPEAE that is clinically unresponsive to relapse-inhibiting fingolimod treatment

Non-active SPMS currently has only one FDA-approved treatment, mitoxantrone, which is rarely prescribed due to significant risk of adverse effects [55]. This lack of treatment options is in part due to a shortage of animal models with neurodegeneration-driven disease progression in which treatments can be tested [12]. While no animal model is a perfect homolog for MS in human patients, several FDA-approved drugs were efficacious in EAE prior to clinical trials, and the EAE model has contributed to our overall understanding of both disease and drug mechanisms of action in MS [for review, see 10]. Therefore, an EAE model that replicates some of the features of SPMS would be an asset to drug discovery.

Fingolimod is efficacious in treating RRMS [8], but not PPMS [29] or non-active SPMS. Therefore, an animal model of SPMS is not expected to respond to fingolimod during the secondary progressive phase of disease. Since EAE in Biozzi mice appears to progress from RREAE to SPEAE based on clinical scores [2, 15, 37], we evaluated the efficacy of daily treatment with fingolimod during RREAE and SPEAE to determine the extent to which SPEAE is independent of newly recruited autoimmune lymphocytes.

**Fig. 1:**
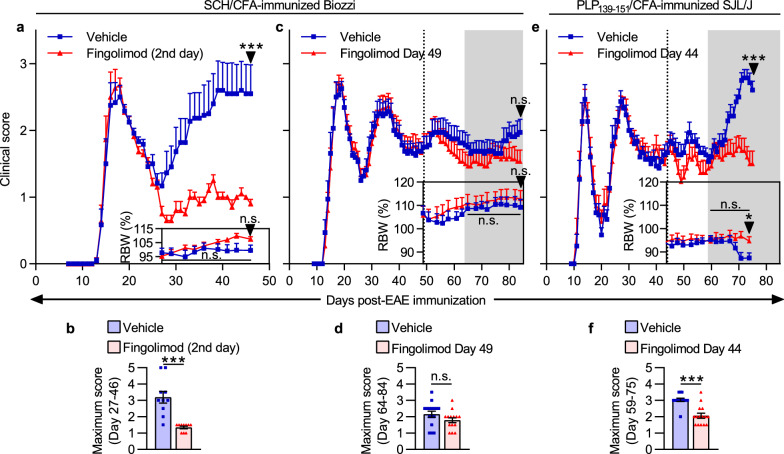
Fingolimod treatment ameliorates RREAE but not SPEAE. **a** Clinical scores, and percent body weights relative to Day -1 (relative body weight, RBW), of Biozzi mice during RREAE. Mice were treated beginning on the 2nd day of disease for each mouse (n = 11–12 mice/group) through Day 45. The study was terminated on Day 46 (arrows). RBW areas under the curves were compared during the relapse period (Day 27 through 46). **c** Clinical scores, and percent body weights relative to Day 0 of Biozzi mice during SPEAE. Mice were treated beginning on Day 49 (dotted line) through Day 83 (n = 15–16 mice/group). The study was terminated on Day 84 (arrows). The gray area represents the efficacy period of treatment (period starting 15 days after treatment onset, i.e., Day 64, through study termination), during which clinical scores and RBW areas under the curves were compared. **e** Clinical scores, and percent body weights relative to Day 0 of SJL/J mice during late RREAE. Mice were treated beginning on Day 44 (dotted line) through Day 74 (n = 15 mice/group). The study was terminated on Day 75 (arrows). The gray area represents the efficacy period of treatment (Day 59 through study termination), during which clinical scores and RBW areas under the curves were compared. **b**, **d**, **f** Maximum EAE scores during the relapse period (**b**) or efficacy period of treatment (**d**, **f**). Data in **a**, **c**, and **e** are shown as mean + SEM and data in **b**, **d**, and **f** are shown as mean ± SEM. Significance was tested using an unpaired two-tailed *t * test (RBW area under the curve and relative end body weight) or two-tailed Mann–Whitney test (end score and maximum score). Results in **c** and **d** are representative of two independent experiments. ***p < 0.001, *p < 0.05, n.s. = not significant

To evaluate the efficacy of fingolimod in RREAE in Biozzi mice, we administered it from the second day of disease in each individual mouse through 45 days from immunization (Day 45; Fig. 1a, b). All scoring of EAE severity was performed by an expert scorer blinded for treatment and for previous scores. As expected, fingolimod significantly suppressed disease during the relapse period (Day 27 through 46) vs. the Vehicle group. We then evaluated the efficacy of fingolimod in Biozzi mice in SPEAE (Fig. 1c, d). We started treatment on Day 49 because in our hands most Biozzi mice develop SPEAE at that time (Additional file 1: Figure S1). Since treatment normally takes time to demonstrate clinical efficacy, we designated the “efficacy period” as the period starting 15 days after treatment onset through study termination.

During the efficacy period, clinical scores of mice in the Fingolimod group were slightly lower than those of mice in the Vehicle group, but there was no significant difference in the mean maximum score (MMS), no significant difference in average scores on any single day, and no significant difference in clinical scores at the end of the study between these groups (Fig. 1c, d). Body weight relative to body weight before EAE induction is reflective of clinical disease severity. The areas under the curves of relative body weight over time (relative to Day 0) were also not significantly different between the Fingolimod and Vehicle groups (Fig. 1c, framed inset graph). This demonstrates that like non-active SPMS in people, SPEAE in Biozzi mice is not dependent on new lymphocyte entry into the CNS.

To confirm that fingolimod can be efficacious in treating relapses even when administered late in the course of disease, we tested the efficacy of fingolimod in PLP_139-151_-immunized SJL/J mice, which unlike Biozzi mice continue to relapse long after disease induction. In contrast to fingolimod’s lack of efficacy in late treatment of Biozzi mice, daily treatment from Day 44 was efficacious at preventing relapses in SJL/J mice (Fig. 1e, f).

### Ibrutinib treatment ameliorates clinical symptoms, suppresses relapses, and disrupts B cell maturation during RREAE in Biozzi mice

B cell-depleting anti-CD20 agents such as ocrelizumab are efficacious in treating RRMS, active SPMS, and PPMS [16, 36, 58], suggesting an important role for B cells in disease pathogenesis. A putative mechanism of action for BTK inhibitors as MS therapies is disruption of B cell receptor signaling and B cell development. Although EAE is largely considered a T cell-dependent disease, B cells are involved in the pathogenesis of some EAE models. PLP_139-151_-induced EAE in SJL/J mice includes formation of tertiary lymphoid tissue containing B cells and B cell chemoattractant CXCL13 in the CNS [3]. Therapeutic inhibition of BTK with evobrutinib in this model has been reported to reduce meningeal inflammation and clinical scores [3]. Since tertiary lymphoid tissue in spinal cords and anti-MOG antibodies in serum were previously reported in Biozzi mice with EAE [37], we hypothesized that their treatment with a BTK inhibitor would ameliorate EAE and disrupt B cell development.

**Fig. 2:**
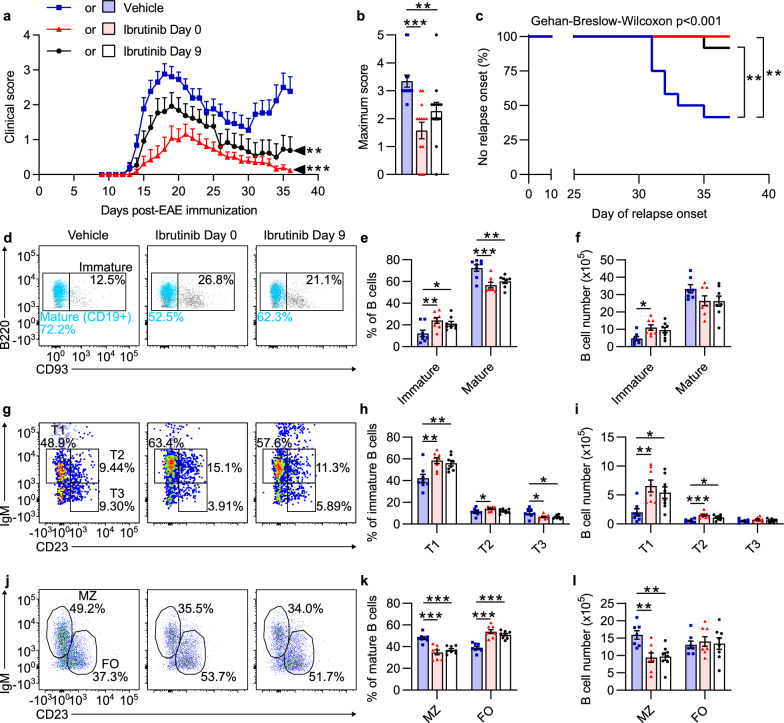
Prophylactic and semi-therapeutic ibrutinib treatment in Biozzi EAE ameliorate clinical symptoms, suppress relapses, and disrupt B cell maturation. Mice were treated daily through Day 35 with vehicle or ibrutinib beginning on Day 0, or ibrutinib beginning on Day 9. Mice treated with ibrutinib from Day 9 received vehicle from Day 0 through Day 8 to control for stress. Clinical EAE analyses (**a**–**c**) were performed on n = 13 mice/group, and flow cytometric analyses (**d–l**) were performed on n = 7–8 representative mice/group from the same experiment. The figure legend in **a** applies to all graphs. **a** EAE clinical scores over time. Arrows indicate the final day of the study (Day 36). **b** Maximum EAE scores for the duration of the study. **c** Kaplan–Meier survival curves for day of relapse onset. **d–l** Spleens were collected and splenocyte suspensions generated on Day 36. **d** Representative dot plots of immature and mature B cells. **e** Proportions of B cells that were immature or mature. **f** Numbers of immature and mature B cells. **g** Representative dot plots with T1, T2, and T3 B cells. **h** Proportions of immature B cells that were T1, T2, or T3 B cells. **i** Numbers of T1, T2, and T3 B cells. **j** Representative dot plots with marginal zone (MZ) and follicular (FO) B cells. **k** Proportions of mature B cells that were MZ or FO B cells. **l** Numbers of MZ and FO B cells. Except in **a** showing mean + SEM and **c** showing total percentage per group over time, data are shown as mean ± SEM. Significance was tested using a Kruskal–Wallis test followed by Dunn’s multiple comparisons test (end score and maximum score), Gehan–Breslow–Wilcoxon tests with *p*values adjusted for multiplicity using the Holm-Sidak approach (time to relapse onset), or one-way ANOVA followed by Dunnett’s multiple comparisons test (flow cytometric analyses). Asterisks denote results of multiple comparisons tests. *p < 0.050, **p < 0.010, ***p < 0.001

To test this hypothesis, we first focused on BTK inhibitor effects in RREAE in Biozzi mice, since BTK inhibitors have demonstrated efficacy in clinical trials with relapsing MS [35, 45]. Mice were treated daily with vehicle beginning on Day 0 (the day of EAE immunization), ibrutinib beginning on Day 0 (prophylactic), or ibrutinib beginning on Day 9 (semi-therapeutic, i.e., after disease induction but prior to symptom onset). These will hereafter be referred to as the Vehicle, Ibrutinib Day 0, and Ibrutinib Day 9 groups, respectively. Compared to the Vehicle group, both Ibrutinib groups developed less severe EAE with significantly lower MMSs, significantly postponed relapse onsets, significantly reduced relapse incidences, and significantly reduced MMSs during the relapse period (Day 31 through Day 36, Fig. 2a–c, Table 1).

**Table 1:** Clinical outcomes of prophylactic and semi-therapeutic ibrutinib treatment in EAE Biozzi mice

Readout	Vehicle (n = 13)	Ibrutinib Day 0 (n = 13)	Post-test *p* value	Ibrutinib day 9 (n = 13)	Post-test p value	Test statistic _(d.f.)_	Overall *p* value
Incidence	13/13	10/13	0.392	12/13	0.999	–	–
Time to onset (median day)	15.0	20.0	**0.002**	15.0	0.055	–	–
Maximum score	3.35 ± 0.77	1.58 ± 1.08	** < 0.001**	2.27 ± 1.13	**0.004**	H_(2)_ = 19.2	< 0.001
Maximum score 1st wave	3.12 ± 0.62	1.54 ± 1.07	** < 0.001**	2.27 ± 1.13	**0.009**	H(_2_) = 17.6	< 0.001
Maximum score 2nd wave	2.65 ± 1.36	0.42 ± 0.70	** < 0.001**	0.77 ± 1.39	**0.001**	H(_2_) = 19.0	< 0.001
Relapse incidence	7/12	0/13	**0.004**	1/12	**0.027**	–	–
Time to relapse onset (median day)	34.0	> 36.0	**0.002**	> 36.0	**0.006**	–	–
End score	2.38 ± 1.52	0.12 ± 0.22	** < 0.001**	0.69 ± 1.42	**0.003**	H_(2)_ = 19.3	< 0.001
Relative end body weight (%)	87.1 ± 11.8	107.6 ± 7.8	** < 0.001**	103.8 ± 9.6	** < 0.001**	F_(2,36)_ = 15.9	< 0.001

Analyses were performed on all mice from the experiment shown in Fig. 2a. Unless otherwise stated, values are shown as mean ± SD. Percent relative end body weight is relative to body weight on Day-1. The 1st wave of EAE was determined to be from Day 9 through 30, and the 2nd wave (relapse period) from Day 31 through 36. Significance for incidence and relapse incidence was tested using two-sided Fisher’s exact tests with *p* values adjusted for multiplicity using the Holm-Sidak approach. Significance for time to onset and time to relapse onset was tested using Gehan–Breslow–Wilcoxon tests between groups with *p* values adjusted for multiplicity using the Holm-Sidak approach. Significance for maximum score and end score was tested using Kruskal–Wallis tests followed by Dunn’s multiple comparisons tests. Significance for percent relative end body weight was tested using a one-way ANOVA followed by Dunnett's multiple comparisons test. Bold p values indicate a significant difference vs. the Vehicle group

BTK inhibition is well known to disrupt B cell maturation. To assess this effect in Biozzi EAE mice, we performed flow cytometry on splenocytes isolated on Day 36 from mice in the Vehicle, Ibrutinib Day 0, and Ibrutinib Day 9 groups (for flow cytometric gating strategies, see Additional file 1: Figure S2). B cell proportions and numbers in spleens from the Ibrutinib groups were similar to those of the Vehicle group (Additional file 1: Table S2). As expected, there were significant shifts in the B cell populations away from mature B cells and toward immature B cells in ibrutinib-treated mice vs. vehicle-treated mice (Fig. 2d–f, Additional file 1: Table S2).

Transitional B cells are immature B cells that have emigrated from the bone marrow and can develop into mature B cells through discrete “transitional” stages. T1 B cells can develop into T2 B cells, which can then develop into mature B cells [28]. T3 B cells (also called An1 B cells) are thought to be anergic cells selected away from this maturation pathway [34]. As expected, the Ibrutinib groups had significantly higher proportions and numbers of T1 B cells than the Vehicle group (Fig. 2g–i, Additional file 1: Table S2). They also had significantly higher numbers of T2 B cells, and the Ibrutinib Day 0 group had a significantly higher proportion of T2 B cells (Fig. 2g–i, Additional file 1: Table S2). The Ibrutinib groups had significantly lower proportions (but similar numbers) of T3 B cells compared to the Vehicle group (Fig. 2g–i, Additional file 1: Table S2).

T2 B cells develop into mature marginal zone (MZ) and follicular (FO) B cells. MZ [27] and FO [25] B cell numbers were reported to be reduced in Xid mice with nonfunctional BTK. In the present study, both Ibrutinib groups had significantly lower proportions and numbers of MZ B cells but significantly higher proportions (and similar numbers) of FO B cells compared to the Vehicle group (Fig. 2j–l, Additional file 1: Table S2), indicating that ibrutinib affects maturation or survival of those cell populations differently.

Mature B cells can further differentiate into memory B cells, or into plasmablasts that can eventually become terminally differentiated plasma cells. There were no significant differences between groups in the proportions or numbers of memory B cells or plasma cells (Additional file 1: Table S2). However, the Ibrutinib Day 0 group had a significantly higher proportion and number of plasmablasts than the Vehicle group (Additional file 1: Table S2).

### Ibrutinib treatment reduces spinal cord pathology during RREAE in Biozzi mice

**Fig. 3:**
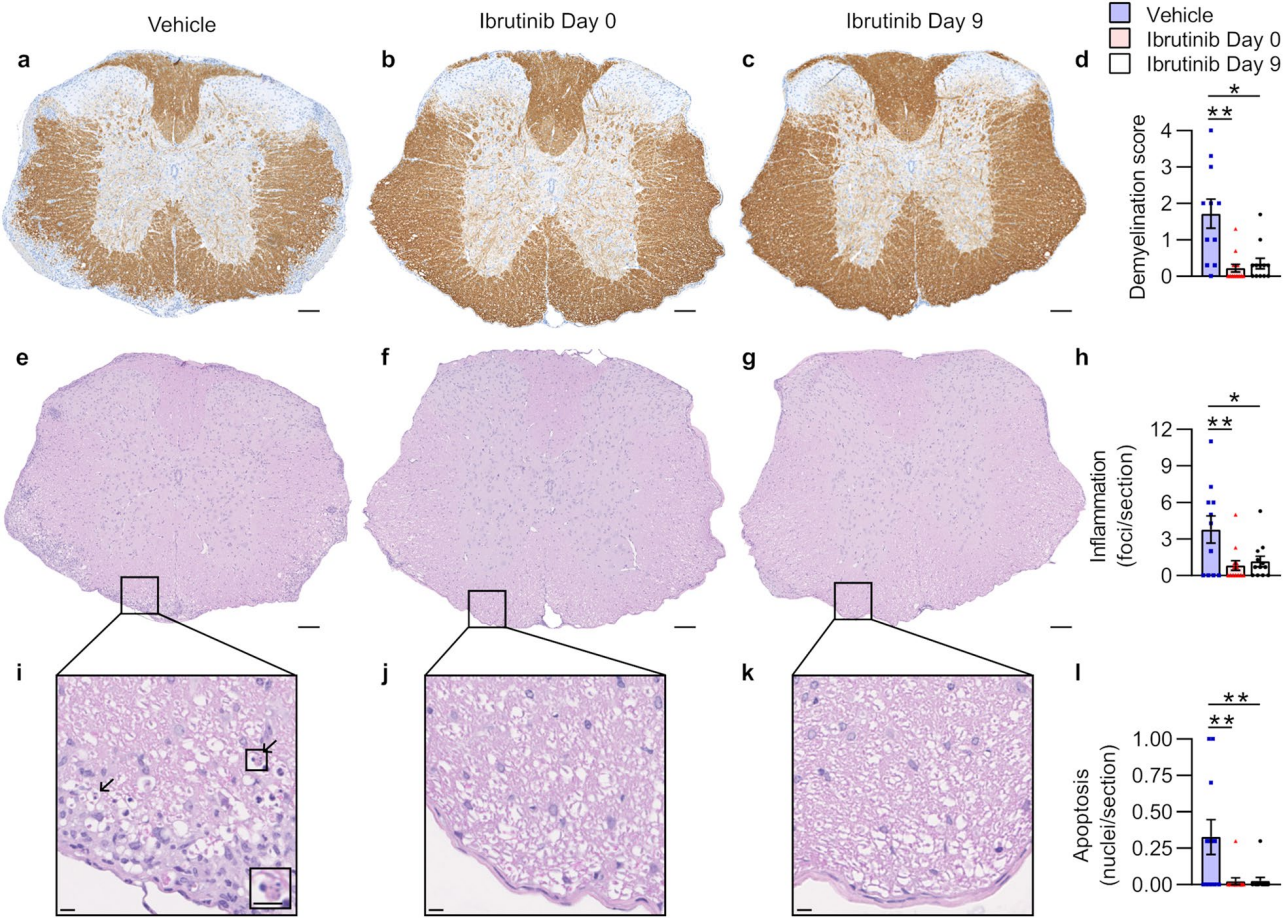
Prophylactic and semi-therapeutic ibrutinib treatment in Biozzi EAE reduce spinal cord demyelination, inflammation, and white matter cell apoptosis. Analyses were performed on all surviving mice (n = 11–13 mice/group) from the experiment shown in Fig. 2a. The figure legend in **d** applies to all graphs. **a-c** Illustrative thoracic spinal cord sections stained with anti-MBP antibody (brown) and hematoxylin (nuclei; blue). Adjustments to gamma were made evenly across these images to better visualize staining. Scale bars, 100 µm. **d** Mean demyelination scores per section, per mouse. **e–g** Illustrative thoracic spinal cord sections stained with hematoxylin (nuclei; blue) and eosin (cytoplasm; pink). Scale bars, 100 µm. **h** Mean inflammation quantified as number of 20+ cell foci per section, per mouse. **i–k** Magnified white matter regions from **e–g** demonstrating inflammatory infiltrates. Arrows in **i** identify apoptotic nuclei, with magnified inset demonstrating an apoptotic nucleus with karyorrhexis. Scale bars, 10 µm. **l** Mean numbers of apoptotic nuclei quantified per section, per mouse. Data in **d**, **h**, and **l** were obtained by taking the mean value of 3 spinal cord regions (cervical, thoracic, and lumbar) from 1 slide per stain, per mouse. Graphed data are shown as mean ± SEM. Significance was tested using a Kruskal–Wallis test followed by Dunn’s multiple comparisons test (**d**) or one-way ANOVA followed by Dunnett’s multiple comparisons test (**h**, **l**). Asterisks denote results of multiple comparisons tests. *p < 0.050, **p < 0.010

Since prophylactic and semi-therapeutic ibrutinib treatment reduced clinical signs of EAE, we examined demyelination, inflammation, and the presence of apoptotic cells in the spinal cord as the pathophysiological correlates of disability. All scoring was performed blind by an expert pathologist (ŽF). In both Ibrutinib groups, the demyelination scores and numbers of inflammatory foci were significantly lower than in the Vehicle group (Fig. 3a–h, Additional file 1: Table S3).

As the primary targets of autoimmunity in MS and EAE, oligodendrocytes undergo apoptosis [32, 42], and prevention of apoptosis is associated with improved clinical outcomes in EAE [32]. While apoptotic cells were rare at the time the mice were euthanized (Day 36), their numbers were significantly lower in the Ibrutinib groups than in the Vehicle group (Fig. 3i–l, Additional file 1: Table S3).

### Ibrutinib treatment reduces clinical and histopathological signs of disease during SPEAE in Biozzi mice

**Fig. 4:**
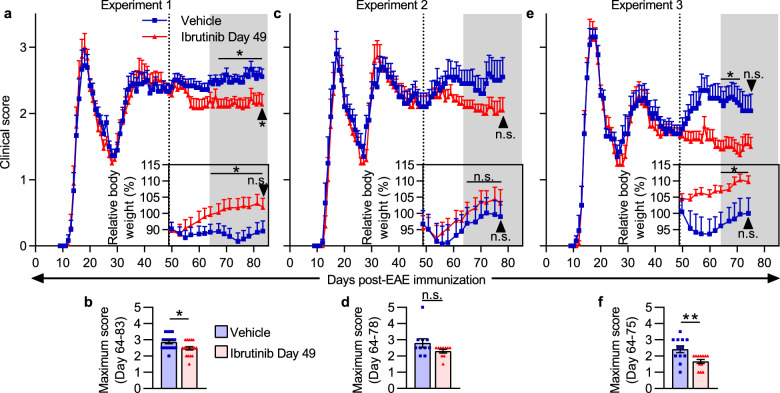
Late therapeutic ibrutinib treatment ameliorates clinical severity of EAE in Biozzi mice. **a–f** SCH/CFA-immunized Biozzi mice were treated with vehicle or ibrutinib daily from Day 49 (dotted lines in **a**, **c**, and **e**) through the day before study termination. Arrows indicate the final day of each experiment (Day 83, Day 78, or Day 75, respectively). Experiment 1 had n = 18 mice/group, Experiment 2 had n = 10 mice/group, and Experiment 3 had n = 13 mice/group. **a**, **c**, **e** Clinical scores, and percent body weights relative to Day-1 (**a**) or Day 0 (**c**, **e**). Gray areas represent the efficacy period of treatment (period starting 15 days after treatment onset, i.e., Day 64, through study termination), during which clinical scores and relative body weight areas under the curves were compared. **b**, **d**, **f** Maximum EAE scores during the efficacy period of treatment. Data in **a**, **c**, and **e** are shown as mean + SEM and data in **b**, **d**, and **f** are shown as mean ± SEM. Significance was tested using an unpaired two-tailed t test (relative body weight area under the curve and relative end body weight), two-tailed Mann–Whitney test (end score and maximum score), or two-tailed Mann–Whitney test followed by the Benjamini and Hochberg procedure (clinical scores on individual days, false discovery rate (FDR) = 5%, bars and asterisks above clinical score graphs indicate FDR-adjusted significance level of individual days). *p < 0.050, **p < 0.010, ***p < 0.001, n.s. = not significant

Peer-reviewed research testing BTK inhibitors in SPEAE has not previously been published. We therefore conducted three independent experiments in which Biozzi mice were enrolled into vehicle- (Vehicle) or ibrutinib-treated (Ibrutinib Day 49) groups on Day 49 after immunization. Mice were enrolled into treatment groups in a balanced manner so that day of onset, EAE score at onset, EAE score at enrollment, and maximum EAE score before treatment started were similar between the groups (Table 2). EAE severity between the groups was compared during the period starting 15 days after enrollment through the end of the study (hereafter termed “efficacy period”). In each experiment (Experiment 1, Experiment 2, and Experiment 3), the Ibrutinib Day 49 group had a lower average clinical score than the Vehicle group during the efficacy period (Fig. 4a–f), with significantly lower MMSs in Experiments 1 (Fig. 4b, Table 2) and 3 (Fig. 4f, Table 2). On multiple days during this period in Experiments 1 and 3, the Ibrutinib Day 49 groups had significantly lower average EAE scores than the Vehicle groups (Fig. 4a, e). In all 3 experiments, the Ibrutinib Day 49 group maintained a higher area under the curve for relative body weight over time during the efficacy period than the Vehicle group, with significant differences in Experiments 1 and 3 (Fig. 4a, c, e, framed inset graphs).

**Table 2:** Clinical outcomes of late therapeutic ibrutinib treatment in EAE Biozzi mice

	Experiment 1			Experiment 2			Experiment 3		
Readout	Vehicle (n = 18)	Ibrutinib Day 49 (n = 18)	Test statistic_(d.f.)_ (*p* value)	Vehicle (n = 10)	Ibrutinib Day 49 (n = 10)	Test statistic_(d.f.)_ (*p* value)	Vehicle (n = 13)	Ibrutinib Day 49 (n = 13)	Test statistic_(d.f.)_ (*p* value)
Balanced for enrollment									
Day of onset	14.9 ± 2.0	14.7 ± 1.7	–	14.6 ± 2.5	14.4 ± 1.3	–	13.7 ± 1.2	13.9 ± 1.4	–
Score at onset	1.11 ± 0.72	1.25 ± 0.69	–	1.05 ± 0.72	1.10 ± 0.57	–	1.31 ± 0.66	1.27 ± 0.75	–
Score at enrollment	2.33 ± 0.42	2.31 ± 0.49	–	2.10 ± 0.66	2.10 ± 0.52	–	1.69 ± 0.48	1.65 ± 0.38	–
Maximum score through Day 49	3.25 ± 0.26	3.28 ± 0.31	–	3.25 ± 0.35	3.20 ± 0.48	–	3.65 ± 0.69	4.12 ± 0.87	–
Readouts of efficacy									
Efficacy period maximum score	2.86 ± 0.45	2.47 ± 0.44	U = 93 **(0.023)**	2.80 ± 0.86	2.30 ± 0.35	U = 30 (0.131)	2.42 ± 0.73	1.67 ± 0.44	U = 29 **(0.009)**
End score	2.56 ± 0.48	2.14 ± 0.64	U = 100 **(0.042)**	2.55 ± 0.93	2.05 ± 0.60	U = 34 (0.197)	2.04 ± 0.86	1.50 ± 0.48	U = 43 (0.088)
Relative end body weight (%)	94.5 ± 13.4	101.9 ± 11.8	t_(34)_ = 1.74 (0.091)	99.0 ± 14.7	103.2 ± 13.3	t_(18)_ = 0.659 (0.518)	100.0 ± 16.6	109.8 ± 6.1	t_(24)_ = 1.91 (0.069)

Analyses were performed on all mice from the experiments shown in Fig. 4. Values are shown as mean ± SD. Percent relative end body weight is relative to body weight on Day -1 (Experiment 1) or Day 0 (Experiments 2 and 3). Significance for efficacy period (period starting 15 days after treatment onset, i.e., Day 64, through study termination) maximum score and end score was tested using two-tailed Mann–Whitney tests. Significance for percent relative end body weight was tested using a two-tailed unpaired *t* test. Bold *p* values indicate a significant difference vs. the Vehicle group

**Fig. 5:**
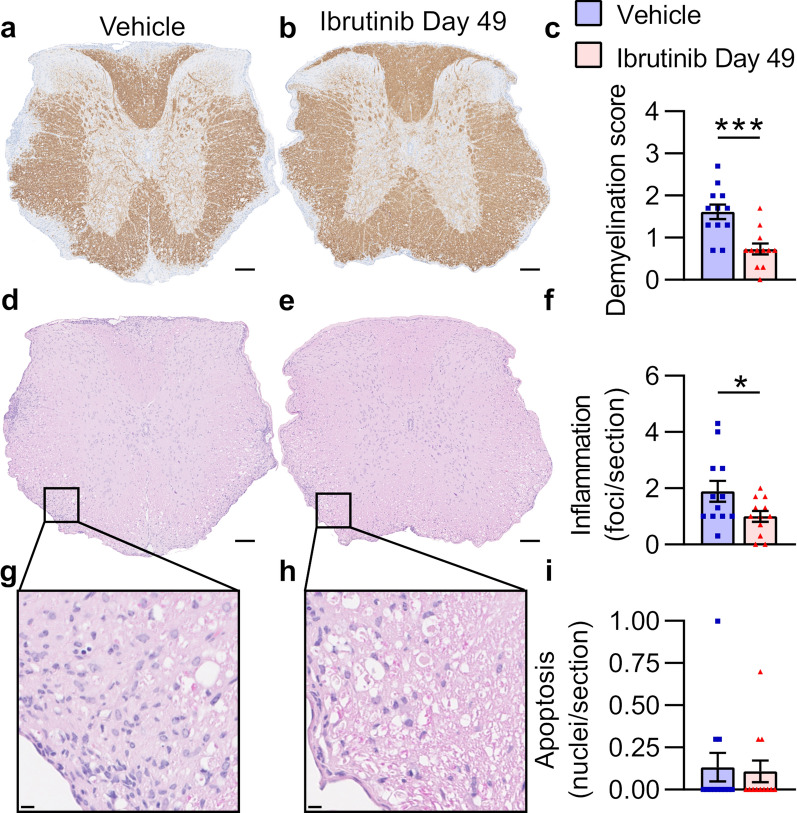
Late therapeutic ibrutinib treatment in Biozzi EAE reduces spinal cord demyelination and inflammation. Analyses were performed on n = 12 representative mice/group from the experiment shown in Fig. 4a. The figure legend in **c** applies to all graphs. **a**, **b** Illustrative thoracic spinal cord sections stained with anti-MBP antibody (brown) and hematoxylin (nuclei; blue). Scale bars, 100 µm. **c** Mean demyelination scores per section, per mouse. **d**, **e** Illustrative thoracic spinal cord sections stained with hematoxylin (nuclei; blue) and eosin (cytoplasm; pink). Scale bars, 100 µm. **f** Mean inflammation quantified as number of 20+ cell foci per section, per mouse. **g**, **h** Magnified white matter regions from **d** and **e** demonstrating inflammatory infiltrates. No apoptotic nuclei with karyorrhexis were identified (for an example of an apoptotic nucleus, see Fig. 3i). Scale bars, 10 µm. **i** Mean numbers of apoptotic nuclei quantified per section, per mouse. Data in **c**, **f**, and **i** were obtained by taking the mean value of 3 spinal cord regions (cervical, thoracic, and lumbar) from 1 slide per stain, per mouse. Graphed data are shown as mean ± SEM. Significance was tested using a two-tailed Mann–Whitney test (**c**) or unpaired two-tailed *t* test (**f**, **i**). *p < 0.050, ***p < 0.001

Spines from all mice in Experiment 1 were analyzed to determine whether improved clinical scores after late therapeutic ibrutinib treatment also resulted in reduced spinal cord pathology (Fig. 5, Additional file 1: Table S4). Both the demyelination scores and the numbers of inflammatory foci were significantly lower in mice from the Ibrutinib Day 49 group than from the Vehicle group (Fig. 5a–f, Additional file 1: Table S4). Very little apoptosis was observed in either group, and there was no significant difference between the groups in numbers of apoptotic cells (Fig. 5g–i, Additional file 1: Table S4).

### Late therapeutic ibrutinib treatment disrupts splenic B cell maturation during SPEAE in Biozzi mice

Similar to observations made after prophylactic and semi-therapeutic ibrutinib treatment (Fig. 2d–l, and Additional file 1: Table S2), spleens from the Ibrutinib Day 49 group had a significantly higher proportion of immature B cells and a significantly lower proportion of mature B cells compared to the Vehicle group (Additional file 1: Table S5). Among immature B cells, proportions of T1 B cells were significantly higher, and proportions of T3 B cells were significantly lower, in the Ibrutinib Day 49 group compared to the Vehicle group (Additional file 1: Table S5). Among mature B cells, proportions and numbers of MZ B cells were significantly lower, and proportions of FO B cells were significantly higher, in the Ibrutinib Day 49 group compared to the Vehicle group (Additional file 1: Table S5). These results all bear resemblance to those in spleens from the prophylactic and semi-therapeutic treatment experiment (Fig. 2d–l, and Additional file 1: Table S2).

We additionally analyzed T follicular helper (TFH) and T follicular regulatory (TFR) cells within the CD4 T cell population. Proportions and numbers of TFH and TFR cells were significantly lower in the Ibrutinib Day 49 group than in the Vehicle group (Additional file 1: Table S5).

Compared to vehicle-treated mice, mice treated prophylactically or semi-therapeutically with ibrutinib had higher numbers of splenocytes and significantly higher numbers of splenic CD4 T cells, while mice treated late therapeutically with ibrutinib had significantly lower numbers of splenocytes and splenic CD4 T cells (Additional file 1: Tables S2 and S5). These contrasting results may be due to the confounding impact of stress-induced corticosteroid production on spleen size [46]. Early in EAE, vehicle-treated mice likely experience more stress than mice treated with an efficacious therapy, resulting in vehicle-treated mice, which have more severe EAE, suffering from more stress and therefore having smaller spleens. The confounding effects of stress on splenocyte numbers are likely to be less pronounced during late chronic disease, as evidenced by higher numbers of splenocytes and splenic CD4 T cells in vehicle-treated mice during the chronic than during the acute phase of EAE (Additional file 1: Tables S2 and S5). During the chronic phase of EAE, the effects of ibrutinib treatment on the numbers of splenocytes and splenic CD4 T are less likely to be obscured by the effects of stress.

**Fig. 6:**
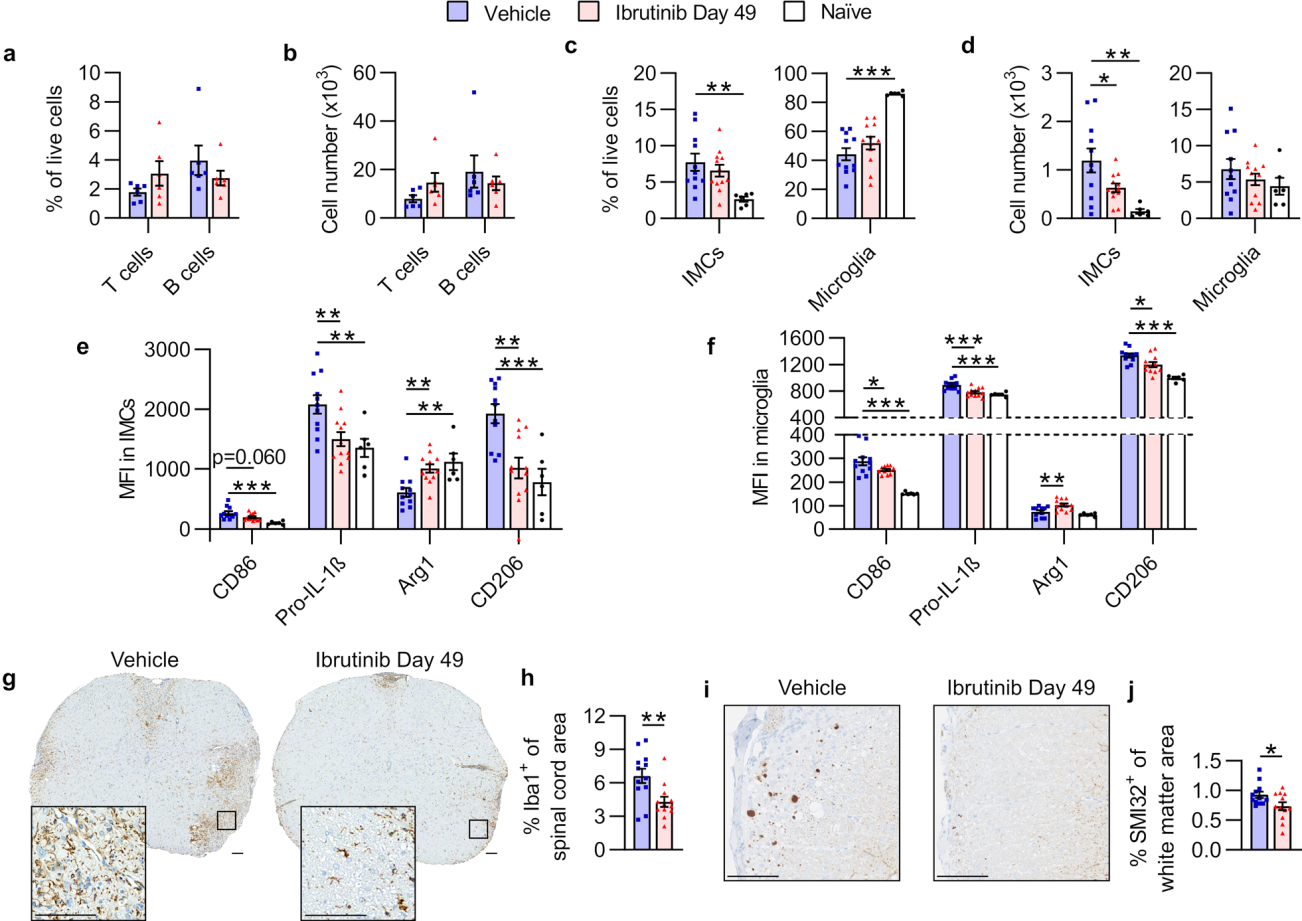
Ibrutinib treatment in Biozzi mice reduces CNS-infiltrating myeloid cell numbers and alters myeloid cell expression of activation markers. **a, b** Analyses were performed on n = 6 representative mice/group from the experiment shown in Fig. 4a. **a** Proportions of CNS-infiltrating lymphocytes that were CD4 T or B cells. **b** Numbers of CNS-infiltrating CD4 T or B cells. **c-f** Analyses were performed simultaneously on n = 6 age-matched naïve mice and n = 11–12 (all surviving) mice/group from the experiment shown in Fig. 4e. **c** Proportions of live cells in spinal cords that were IMCs or microglia. **d** Numbers of spinal cord IMCs or microglia. **e**,** f** Median fluorescence intensity (MFI) of activation markers in spinal cord IMCs or microglia. **g-j** Analyses were performed on n = 12 representative mice/group from the experiment shown in Fig. 4a. **g** Illustrative cervical spinal cord sections stained with anti-Iba1 antibody (brown) and hematoxylin (nuclei; blue). Magnified insets demonstrate Iba1^+^ areas in white matter. Scale bars, 100 µm. **h** Mean proportions of Iba1^+^ areas in whole spinal cord sections per mouse. **i** Illustrative cervical spinal cord white matter stained with anti-SMI32 antibody (brown) and hematoxylin (nuclei; blue). Both images are from the same white matter area (ventrolateral). Scale bars, 100 µm. **j** Mean proportions of SMI32^+^ areas in spinal cord white matter per mouse. Adjustments to input levels were made evenly across images in **g**, and evenly across images in **i**, to better visualize staining. Data in **h** and **j** were obtained by taking the mean value of 3 spinal cord Sects. (1 each of cervical, thoracic, and lumbar regions) per stain, per mouse. Graphed data are shown as mean ± SEM. Significance was tested using an unpaired two-tailed *t* test (**a**, **b**, **h**, and **j**) or one-way ANOVA followed by Dunnett's multiple comparisons test (**c–f**). *p < 0.050, **p < 0.010, ***p < 0.001

To determine what changes in CNS-infiltrating lymphocytes accompanied ibrutinib treatment, we performed flow cytometry on cells isolated from the CNS (brain and spinal cord combined). There were no significant differences between the groups in the numbers or proportions of CNS-infiltrating CD4 T or B cells (Fig. 6a, b, Additional file 1: Table S6).

### Late therapeutic ibrutinib treatment alters infiltrating myeloid cell (IMC) and microglia activation and reduces axonal damage in the spinal cord during SPEAE in Biozzi mice

In addition to its expression by B cells, BTK is expressed by cells of the myeloid lineage, where it mediates processes including cytokine production, phagocytosis, and survival [6, 20, 24, 33]. Considering this, we explored the effects of late therapeutic ibrutinib treatment in Biozzi EAE mice on CD45^hi^CD11b^+^ spinal cord IMCs and CD45^int^CD11b^+^ resident microglia using flow cytometry. While proportions of myeloid cells in spinal cords were not significantly affected by late therapeutic ibrutinib treatment, the number of IMCs was significantly lower in the Ibrutinib Day 49 group than in the Vehicle group (Fig. 6c, d, Additional file 1: Table S7).

To determine whether BTK inhibition affected myeloid cell activation, we measured expression of CD86, pro-IL-1β (precursor for pro-inflammatory cytokine IL-1β), TNF, and iNOS, which are generally expressed by pro-inflammatory or activated myeloid cells, in both IMCs and microglia. In both cell types, expression of CD86 and pro-IL-Iβ was either significantly or close to significantly lower in the Ibrutinib Day 49 group than in the Vehicle group, with expression of pro-IL-1β in the Ibrutinib Day 49 group approaching that of naïve mice (Fig. 6e, f, Additional file 1: Table S7). We also measured expression of Arg1, CD206, and IL-10, which are generally expressed by immunosuppressive or reparative myeloid cells. No significant differences were observed in expression of IL-10 between myeloid cells from the Vehicle and the Ibrutinib Day 49 groups. Expression of Arg1 was significantly higher in both IMCs and microglia from the Ibrutinib Day 49 group compared to the Vehicle group, with expression in microglia from the Ibrutinib Day 49 group surpassing that of microglia from naïve (non-immunized) mice (Fig. 6e, f, Additional file 1: Table S7). In contrast, expression of CD206 was significantly lower in the Ibrutinib Day 49 group than in the Vehicle group (Fig. 6e, f, Additional file 1: Table S7).

Considering these changes in activation markers of myeloid cells, we also examined spinal cord myeloid cells by immunohistochemistry. Microglia undergo morphological changes during activation, going from a ramified to more amoeboid shape with shorter and thicker processes [9]. Expression of ionized calcium binding adaptor molecule 1 (Iba1) is upregulated in activated macrophages/microglia during MS [59] and EAE [5]. Spinal cords from the Vehicle group had many Iba1^+^ myeloid cells with enlarged cell bodies and thick processes, as typical of mice with EAE (Fig. 6g). In contrast, spinal cords from the Ibrutinib Day 49 group had fewer visibly Iba1^+^ myeloid cells, with most myeloid cells having thinner processes than observed in the Vehicle group (Fig. 6g). Additionally, the proportion of tissue area stained with anti-Iba1 antibody was significantly smaller in the Ibrutinib Day 49 group than in the Vehicle group (Fig. 6h, Additional file 1: Table S8).

Axonal degeneration correlates with irreversible neurological disability and is a major contributor to disease progression in MS [4]. Therefore, an important factor in treating progressive MS is prevention of axonal damage. Anti-SMI32 antibody binds to non-phosphorylated neurofilament, which is found in damaged axons [53]. The proportion of tissue area that stained with anti-SMI32 antibody was significantly smaller in the Ibrutinib Day 49 group than in the Vehicle group (Fig. 6i, j, Additional file 1: Table S8). These data show that in addition to regulating activation of myeloid cells, BTK inhibition results in less axonal damage, which may facilitate recovery from disease.

### The highly selective, CNS-penetrant BTK inhibitor GB7208 is efficacious at reducing clinical disease in SPEAE in Biozzi mice

**Fig. 7:**
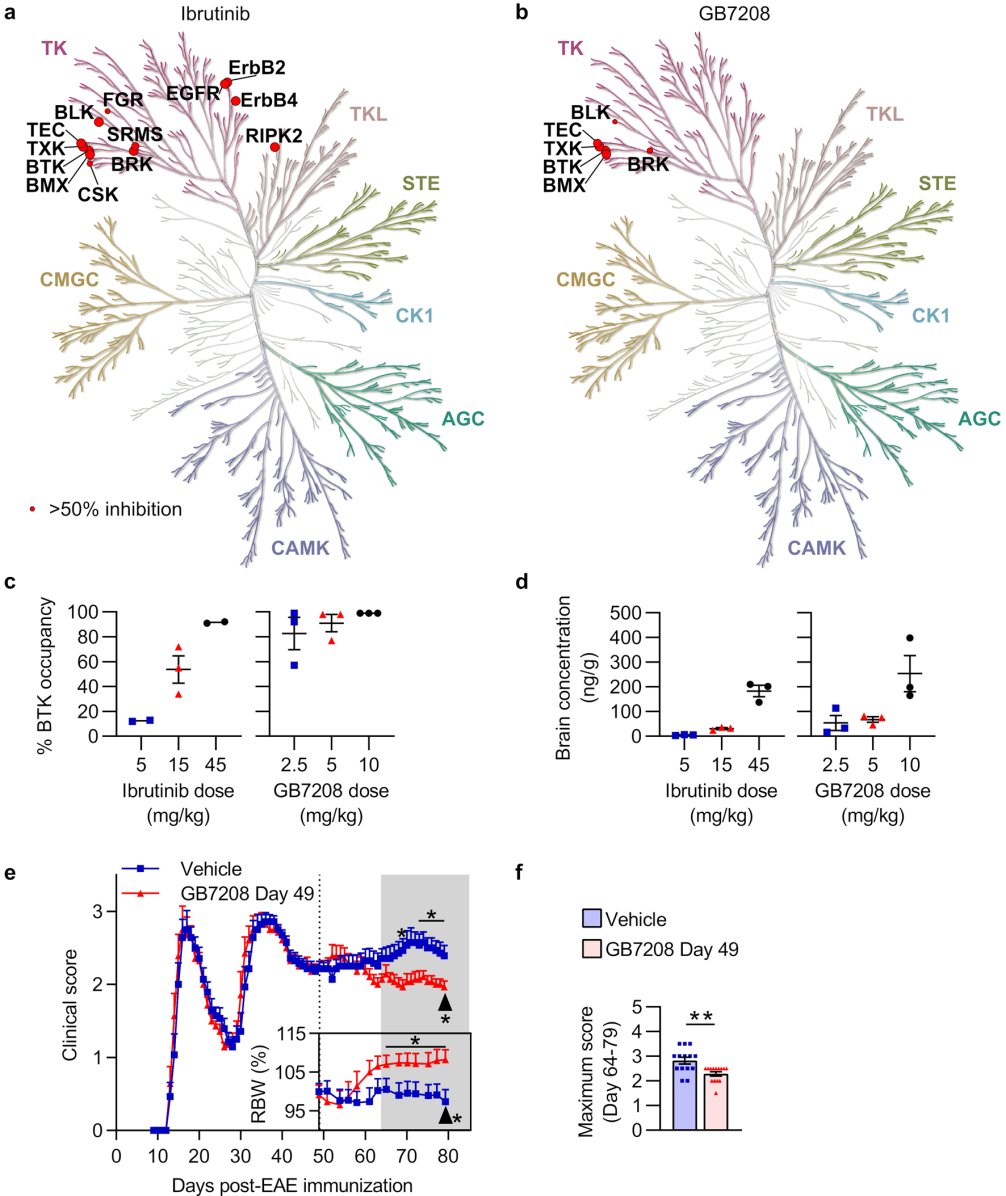
Novel BTK inhibitor GB7208 demonstrates greater selectivity and CNS target occupancy than ibrutinib and ameliorates SPEAE in Biozzi mice. **a**, **b** Kinases in a 357-kinase assay inhibited greater than 50% at 1 µM concentrations of ibrutinib (**a**) or GB7208 (**b**) with 1 mM ATP. Larger red circles indicate greater inhibition. Kinome trees were generated using KinMap [11], and tree illustrations were reproduced courtesy of Cell Signaling Technology, Inc. (www.cellsignal.com). **c-d** Target occupancy (**c**) and BTK inhibitor concentration (**d**) in brains from n = 2–3 naïve C57BL/6J mice/group dosed orally, daily, for a total of 3 days with either ibrutinib or GB7208. Tissue was collected 1 h after the final dose. Error bars are not shown for groups with n = 2 (5 and 45 mg/kg ibrutinib groups for % BTK occupancy). Data for ibrutinib and GB7208 are from separate experiments. **e**, **f** SCH/CFA-immunized Biozzi mice were treated with vehicle or 5 mg/kg GB7208 (n = 14 mice/group) daily from Day 49 (dotted line in **e**) through Day 79. Arrows indicate the final day of the study (Day 79). **e** Clinical scores, and percent body weights relative to Day 0 (relative body weight, RBW). Gray area represents the efficacy period of treatment (period starting 15 days after treatment onset, i.e., Day 64, through study termination), during which clinical scores and RBW areas under the curves were compared. **f** Maximum EAE scores during the efficacy period of treatment. Data in **e** are shown as mean + SEM, and data in **c**, **d**, and **f** are shown as mean ± SEM. Significance was tested using an unpaired two-tailed t test (RBW area under the curve and relative end body weight), two-tailed Mann–Whitney test (end score and maximum score), or two-tailed Mann–Whitney test followed by the Benjamini and Hochberg procedure (clinical scores on individual days, false discovery rate (FDR) = 5%, bars and asterisks above clinical score graph indicate FDR-adjusted significance level of individual days). *p < 0.050, **p < 0.010

Although the first generation BTK inhibitor ibrutinib was efficacious in treating SPEAE, ibrutinib lacks the selectivity of some newer generation BTK inhibitors, such as GB7208, a CNS-penetrant, pre-clinical BTK inhibitor. In a kinome scan against 357 kinases at 1 µM with 1 mM ATP, ibrutinib inhibited nine non-Tec family kinases with greater than 50% inhibition (Fig. 7a), while GB7208 inhibited only two non-Tec family kinases, BRK/PTK6 and BLK, with greater than 50% inhibition (Fig. 7b). The target occupancy of BRK/PTK6 and BLK by GB7208 was further de-risked in cell-based assays (Additional file 1: Figure S3). GB7208 had similar potency to ibrutinib in several in vitro assays based on IC_50_, and a fivefold faster rate of inactivation (*k*_*inact*_/*K*_*i*_) of BTK in human whole blood (Additional file 1: Table S9). GB7208 inhibited proliferation of human B cells in vitro with an IC_50_ of 0.5 nM ± 0.1 nM and reduced BCR-mediated calcium influx in Ramos B cells with an IC_50_ of 7.4 nM ± 2.6 nM (mean ± SD; Additional file 1: Table S9). GB7208 had superior CNS penetrance to ibrutinib in naïve C57BL/6J mice with intact blood–brain barriers (Fig. 7c, d). During SPEAE, Biozzi mice treated with GB7208 had significantly lower maximum scores, significantly lower end scores, significantly greater (improved) areas under the curve for relative body weight, and significantly higher end body weights compared to mice treated with vehicle (Fig. 7e, f, Table 3).

**Table 3:** Clinical outcomes of late therapeutic GB7208 treatment in EAE Biozzi mice

Readout	Vehicle (n = 14)	GB7208 Day 49 (n = 14)	Test statistic_(d.f.)_ (p value)
Balanced for enrollment			
Day of onset	14.3 ± 1.4	14.1 ± 1.6	–
Score at onset	1.29 ± 0.67	1.43 ± 0.51	–
Score at enrollment	2.21 ± 0.38	2.21 ± 0.38	–
Maximum score through Day 49	3.18 ± 0.32	3.25 ± 0.26	–
Readouts of efficacy			
Efficacy period maximum score	2.82 ± 0.50	2.29 ± 0.32	U = 40 **(0.005)**
End score	2.39 ± 0.53	1.96 ± 0.31	U = 49.5 **(0.017)**
Relative end body weight (%)	97.4 ± 11.6	108.2 ± 9.6	t_(26)_ = 2.69 **(0.012)**

Analyses were performed on all mice from the experiment shown in Fig. 7e. Values are shown as mean ± SD. Percent relative end body weight is relative to body weight on Day 0. Significance for efficacy period (period starting 15 days after treatment onset, i.e., Day 64, through study termination) maximum score and end score was tested using two-tailed Mann–Whitney tests. Significance for percent relative end body weight was tested using a two-tailed unpaired t test. Bold p values indicate a significant difference vs. the Vehicle group

## Discussion

Currently, non-active SPMS presents a clinical challenge with few treatment options. This is partly due to a lack of understanding of the pathological mechanisms of progressive MS, and to the lack of suitable animal models [12]. The hallmark of non-active SPMS is progression independent of relapse activity (PIRA). While the efficacy of fingolimod has not been evaluated in a clinical trial for SPMS, natalizumab which, like fingolimod blocks entry of peripheral lymphocytes into the CNS and inhibits clinical relapses, has not shown convincing evidence of efficacy in reducing disease progression in patients with SPMS [21], confirming independence of disease progression from newly recruited CNS-infiltrating cells. In the present study, we postulated that a model of SPMS should be independent of newly recruited CNS-infiltrating lymphocytes, but still treatable by therapies that could be efficacious in SPMS. We demonstrated fingolimod’s lack of efficacy in clinically improving EAE in Biozzi mice when administered starting 49 days after immunization. This indicates that as in SPMS, progression of SPEAE in Biozzi mice is largely independent of newly recruited lymphocytes.

The pathology of Biozzi EAE also bears some similarity to that of SPMS, including meningeal tertiary lymphoid tissue rich with clusters of B cells, CD8^+^ T cells, and Iba1^+^ microglia/macrophages associated with subpial demyelinated plaques with axonal loss [37]. There are important differences between SPEAE in Biozzi mice and SPMS in human patients, however, that represent limitations of the Biozzi EAE model. These include (1) the absence of slowly expanding lesions which consist of an inactivated, demyelinated center with a rim of Iba1^+^ microglia/macrophages [41], and (2) high titers of anti-MOG antibody in serum [37], which play a role in MOG antibody disease (MOGAD) but are not thought to be involved in MS [18]. The extent of similarity between EAE in Biozzi mice and MOGAD has yet to be determined. Unlike the secondary progressive phase of EAE in Biozzi mice, there is no known progressive phase of MOGAD. Additionally, initial EAE development and development of EAE relapses in Biozzi mice do not rely on B cells or the humoral response, as evidenced by the failure of B cell depletion with anti-CD20 antibody to inhibit clinical disease symptoms [48]. Whether anti-MOG antibody is involved in ongoing pathology during SPEAE, and the extent of its involvement, requires further exploration.

While selecting the target for treatment of SPEAE, we considered that: (a) oligoclonal bands in cerebrospinal fluid are a marker of MS and represent Ig produced by clonally expanded intrathecal B cells [38], (b) B cell depletion with ocrelizumab is the only approved therapy for PPMS [14], (c) the presence of ectopic B cell follicles is associated with severe SPMS [30], and (d) activated myeloid cells (microglia and macrophages) are associated with expanding lesions in SPMS [41]. BTK inhibitors are promising potential therapies for MS, and possibly for non-active SPMS, due to their ability to disrupt B cell maturation without depleting all B cells [52], and due to their ability impact myeloid cell activation and function [6, 20, 24, 33]. Indeed, several phase 3 clinical trials are currently under way evaluating efficacy of BTK inhibitors in various forms of MS, including non-active SPMS (HERCULES/NCT04411641).

We therefore tested the efficacy of BTK inhibitors in Biozzi EAE. Ibrutinib treatment starting at the time of immunization, 9 days after immunization, and 49 days after immunization with SCH/CFA was efficacious at reducing EAE severity. Similarly, treatment with the more selective, CNS-penetrant BTK inhibitor, GB7208, starting 49 days after immunization was efficacious at reducing EAE severity. Like ibrutinib, GB7208 inhibited B cell proliferation and was previously shown to inhibit myeloid cell functions (poster presented at ACTRIMS Forum 2023, Feb. 23–25: Yusuf I, et al. P406). GB7208 dosed at 5 mg/kg had similar target occupancy in the brain to ibrutinib dosed at 45 mg/kg in naïve C57BL/6J mice, despite having a lower brain concentration (67.8 ng/g for 5 mg/kg GB7208 vs. 183 ng/g for 45 mg/kg ibrutinib). While the efficacies of 50 mg/kg ibrutinib and 5 mg/kg GB7208 in ameliorating EAE were not directly compared in the same experiment, the efficacy of each drug at those respective doses appeared to be at least similar compared to their respective negative control groups.

In SPMS, myeloid cells are associated with expanding lesions and ongoing demyelination even in the absence of acute inflammation [41]. Meningeal ectopic B cell follicles in SPMS patients are associated with increased microglia activation and loss of neurites compared to SPMS patients without ectopic B cell follicles [30]. This compartmentalized inflammation and neurite degeneration is considered a major driver of PIRA and a reason for the resistance of progressive MS to treatment with anti-inflammatory drugs [44]. After late therapeutic ibrutinib treatment during SPEAE in Biozzi mice, we found reduced spinal cord pathology including less demyelination, fewer inflammatory foci, fewer IMCs, less Iba1^+^ tissue area, and less axonal damage compared to vehicle-treated mice. Results of our immunohistochemical and flow cytometric analyses show correlation between the reduced activation of myeloid cells in spinal cords and therapeutic efficacy of ibrutinib treatment. This is consistent with the observation that in non-relapsing MS patients, activation of myeloid cells in perilesional normal-appearing white matter—as measured by PET imaging of radioligand binding to translocator protein (TSPO)—is a significant predictor of disease progression [50]. Together, these results, alongside improved clinical outcomes, suggest that BTK inhibition could help mitigate PIRA in SPMS by reducing compartmentalized inflammation.

The downregulation of homeostatic genes in myeloid cells during MS is thought to contribute to disease progression [59]. In addition to reduced spinal cord pathology, we found that in both IMCs and tissue-resident microglia, expression of inflammatory markers CD86 and pro-IL-1β and expression of anti-inflammatory marker CD206 were reduced, while expression of anti-inflammatory marker Arg1 was increased, in ibrutinib-treated mice compared to vehicle-treated mice during SPEAE. Except for higher expression of Arg1 by microglia, all other differences brought expression of these markers closer to those observed in naïve mice, suggesting more homeostatic gene expression by myeloid cells after ibrutinib treatment than after vehicle treatment. Since BTK signaling in myeloid cells is involved in their cytokine production, phagocytosis, and survival [6, 20, 24, 33], our observations may be a result of direct effects of BTK inhibition on myeloid cells. For instance, BTK signaling in myeloid cells is involved in activation of the NOD-like receptor protein 3 (NLRP3) inflammasome, whose activation increases expression of pro-IL-1β [17, 31]. As mentioned above, inhibition of BTK during SPEAE in Biozzi mice reduced myeloid cell expression of pro-IL-1β in the present study.

Our observations could also result from indirect effects of B cells on myeloid cells. For example, BTK inhibition in PLP_139-151_/CFA-immunized SJL/J mice was previously shown to reduce the formation of meningeal ectopic lymphoid structures [3]. Since these structures are associated with increased myeloid cell activation [30], their dissolution could reduce compartmentalized inflammation. Reduced B cell activation and differentiation after BTK inhibition may also reduce immunoglobulin production, as evidenced by immunoglobulin deficiency in BTK-deficient mice and people [25]. This would indirectly affect FcγR-mediated activation of, and phagocytosis by, microglia [51, 54].

## Conclusions

Overall, our results demonstrate both the suitability of EAE in Biozzi mice as a model of secondary progressive autoimmune demyelination, and the ability of BTK inhibition to suppress disease progression. By modulating B cells and myeloid cells with a CNS-penetrant BTK inhibitor, it may be possible to overcome the problem of compartmentalized inflammation. This would reduce neurodegeneration and PIRA, leading to slower disease progression and allowing reparative mechanisms to occur.

## Supplementary Information

**Additional file 1**: Supplementary methods, tables, and figures.

**Additional file 2**: Underlying data for all figures and tables.

## Data Availability

All data generated or analyzed during this study are included in this published article and in Additional file 2.

## Abbreviations

BTK: Bruton's tyrosine kinase
CFA: Complete Freund's adjuvant
CNS: Central nervous system
EAE: Experimental autoimmune encephalomyelitis
FDA: United States Food and Drug Administration
FO: Follicular
IMC: Infiltrating myeloid cell
MMS: Mean maximum score
MOG: Myelin oligodendrocyte glycoprotein
MOGAD: Myelin oligodendrocyte glycoprotein antibody disease
MZ: Marginal zone
MS: Multiple sclerosis
PIRA: Progression independent of relapse activity
PPMS: Primary progressive multiple sclerosis
RREAE: Relapsing–remitting experimental autoimmune encephalomyelitis
RRMS: Relapsing–remitting multiple sclerosis
SCH: Spinal cord homogenate
SPEAE: Secondary progressive experimental autoimmune encephalomyelitis
SPMS: Secondary progressive multiple sclerosis
TFH: T Follicular helper
TFR: T Follicular regulatory
