# Supplementary material for: Bruton’s tyrosine kinase inhibition reduces disease severity in a model of secondary progressive autoimmune demyelination
Source: Acta Neuropathol Commun. 2023 Jul 12;11:115. doi: 10.1186/s40478-023-01614-w (PMC10337138; doi:10.1186/s40478-023-01614-w)
Supplement: Supplementary file 1 — Additional file 1: Supplementary methods, tables, and figures. [file 40478_2023_1614_MOESM1_ESM.pdf]

ADDITIONAL FILE 1 (SUPPLEMENTARY INFORMATION)

Bruton's tyrosine kinase inhibition reduces disease severity in a model of secondary progressive autoimmune demyelination

Kirsten Scarlett Evonuk[1], Sen Wang[1], Josh Mattie[1], CJ Cracchiolo[1], Reine Mager[1], Željko Ferenčić[1], Ethan Sprague[1], Brandon Carrier[1], Kai Schofield[1], Evelyn Martinez[1], Zachary Stewart[1], Tara Petrosino[1], Gregory Andrew Johnson[1], Isharat Yusuf[2], Warren Plaisted[2], Zachary Naiman[2], Timothy Delp[1], Laura Carter[2], Suzana Marušić[1]

[1]Hooke Laboratories, Lawrence, MA, USA
[2]Gossamer Bio, San Diego, CA, USA

Corresponding authors:
Kirsten Scarlett Evonuk
Hooke Laboratories, LLC
439 South Union Street
Lawrence, MA 01843 USA
Phone: +1 617 475 5114
Fax: +1 617 395 1352
k.evonuk@hookelabs.com

Suzana Marušić
Hooke Laboratories, LLC
439 South Union Street
Lawrence, MA 01843 USA
Phone: +1 617 475 5114
Fax: +1 617 395 1352
s.marusic@hookelabs.com

SUPPLEMENTARY METHODS

Ramos cell calcium flux assay

Inhibition of BCR signaling was assessed in vitro using Ramos cells (cell line ATCC CRL-1596) in a 384-well fluorometric imaging plate reader (FLIPR^{TM}) assay measuring anti-IgM-mediated intracellular calcium flux in response to receptor activation. Cells cultured in RPMI-1640, 10% FBS (Hyclone, cat. no. SH30406), 100 U/mL penicillin, and 100 µg/mL streptomycin were maintained at 10^5 to 10^6 cells/mL and transferred to RPMI-1640, 1% FBS, 100 U/mL penicillin, and 100 µg/mL streptomycin the day before the assay. Cells were dye-loaded (FLIPR Calcium 6, Molecular Devices, cat. no. R8191) in the presence of 2.5 mM probenecid (Invitrogen), seeded at 3 x 10^4 cells/well into poly-D-lysine coated plates (Labcyte, cat. no. PP-0200), and incubated at 37 °C for 2 hours followed by 25 °C for 15 minutes. Compounds were serially diluted and prepared at 3x the final assay concentration in 20 mM HEPES, 0.1% BSA (Merck, cat. no. 12659), and Hank's buffered saline solution, pH 7.4. Fluorescence values were obtained (using Molecular Devices FLIPR^{TM} Tetra: excitation 485 nm/emission 515 nm, collected over 160 seconds using 1 second intervals) immediately following addition of compounds. Plates were then incubated at 25 °C for 1 hour in the dark prior to the addition of anti-IgM (Jackson ImmunoResearch, cat. no. 109-006-129) at the EC_{80}, and fluorescence again measured. The maximum minus minimum fluorescence was calculated for each sample and transformed to percent inhibition relative to control wells (without compound treatment, with or without anti-IgM stimulation). IC_{50} values were determined using a 3-parameter logistic model (XLFit, Microsoft Excel).

Cellular kinetic assays

Time- and dose-dependent inhibition of BTK in whole blood cells or brain cell lysates following ex vivo treatment with BTK inhibitors was measured utilizing a biotinylated probe binding to unoccupied BTK active sites and detecting it using 96-well streptavidin-coated ELISA plates (R&D Systems, cat. no. CP004). Serially diluted compounds in PBS, at 10x the final assay concentration, were added to whole blood from healthy human donors (blood was collected in tubes with sodium heparin; StemExpress, cat. no. PBNAH010F) or mouse and incubated at 37 °C for 5, 10, 15, 30, or 60 minutes. Cell lysis buffer (Cell Signaling Technology, cat. no. 9083S) containing protease inhibitors (ThermoFisher, cat. no. 78440) and 0.4 µM BTK active site probe was added (1:1, v/v) and samples frozen at -80 °C. For brain cell lysate, mouse brain tissue (left hemisphere) was dissociated (Miltenyi Biotec gentleMACS) in 750 µL lysis buffer containing protease inhibitors, debris removed by centrifugation, and samples gently rotated overnight at 4 °C. Compounds (10x in PBS) were added to brain lysate, incubated at 37 °C for 5, 10, 15, 30, or 60 minutes, BTK active site probe was added to a final concentration of 0.2 µM, and samples were shaken at ambient temperature for 60 minutes.

Thawed blood cell lysates (1:10) or freshly prepared brain lysates (1:2) were diluted in PBS, 0.05% Tween 20, 1% BSA, containing protease inhibitors and anti-BTK (Cell Signaling Technology, cat. no. 8547S) at a final dilution of 1:1000 and incubated at

ambient temperature for 90 minutes. After washing (PBS + 0.05% Tween 20), 1:2500 donkey anti-rabbit IgG detection antibody (Jackson ImmunoResearch, cat. no. 711-005-152) in PBS, 0.05% Tween 20, 1% BSA was added and incubated at ambient temperature for 1 hour. Tetramethylbenzidine substrate (ThermoFisher, cat. no. 34029) was added after washing and incubated for 5 minutes in the dark before stopping the reaction. Absorbance at 450 nm and 570 nm (correction wavelength) to quantify unoccupied BTK active sites was measured using a BMG PHERAstar FSX microplate reader (BMG Labtech). Absorbance data was fit to a one phase decay model to obtain the rate constant for inhibition, k_{obs} (GraphPad Prism). Rate constants (k_{obs}) for each compound concentration were then re-analyzed against concentrations and fit to a hyperbolic equation (Michaelis-Menten model). Using this model, the best-fit values for V_{max} (k_{inact}) and K_{m} (K_i) were used to determine a second order rate constant for the inactivation in whole blood or brain lysates using the formula (k_{inact}/K_i)*10,000, which is then represented as the calculated BTK inactivation rate constant (k_{inact}/K_i)10^{-4}nM^{-1}min^{-1}.

BTK kinase enzymatic assay

BTK cell-free enzymatic assays were conducted in a 384-well LabChip EZ Reader (Perkin Elmer, cat. no. 122919) microfluidic assay using protein kinase 5-FAM fluorescent substrates. Human kinases were purchased from Carna Biosciences (BTK cat. no. 08-180). For IC_{50} determination, compounds were serially diluted and prepared at 1000x the final assay concentration in DMSO and reacted with substrate and kinase. All assay conditions included 1 mM ATP, 1.50 µM fluorescent peptide substrate, 0.55-2.67 nM kinase in 50 mM HEPES, 0.2 mM DTT, 0.01% Brij-35, 1 mM EGTA, and 0.05% BSA. Reactions contained 10 mM $MgCl_2$ as cofactor. FL Peptide2 (Perkin Elmer, cat. no. 760430) was the substrate for BTK. Reactions were stopped after 90 minutes, fluorescence was measured, and IC_{50} values were determined using a 4-parameter logistic model (XLFit, Microsoft Excel). Fluorescence units were transformed to percent inhibition relative to control wells without compound treatment.

BRK/PTK6 and BLK NanoBRET assays

HEK293 cells were transfected with BLK-NanoLuc Fusion Vector (Promega, cat. no. NV2461) or BRK/PTK6-NanoLuc Fusion Vector (Promega, cat. no. NV1941). The day after transfection, cells were re-suspended in Opti-MEM medium and plated into a 384-well plate at a density of 7000 cells/well. The cells were exposed to GB7208 using a range of concentrations in the presence of 1 µM (for BRK) or 0.3 µM (for BLK) tracer K-4 (Promega, cat. no. N2520) for 2 hours. After exposure, the Nano-Glo substrate (Promega, cat. no. N1571) was added and NanoBRET bioluminescence (donor:acceptor ratio) measured on a Synergy Neo2 instrument (Agilent Technologies). Percentage of BTK occupancy by GB7208 was calculated as percentage of residual bioluminescence (donor:acceptor ratio) in the presence of compound vs. without compound.

SUPPLEMENTARY FIGURES

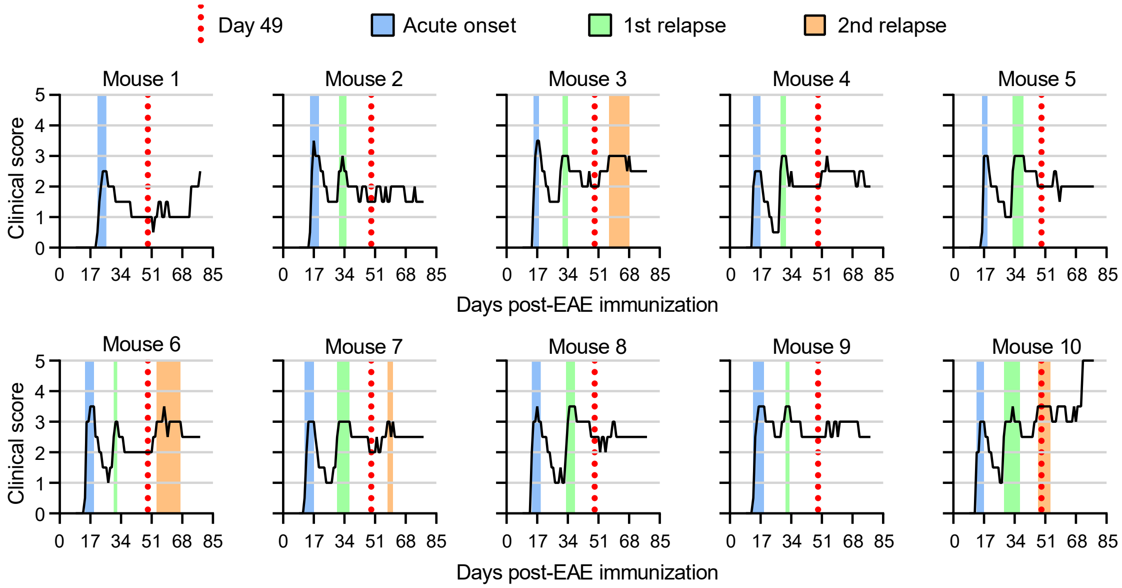

Figure S1 EAE in Biozzi mice immunized with SCH/CFA begins as a relapsing-remitting disease that develops into a secondary progressive disease. Mice were immunized with 0.75 mg SCH and 0.07 mg *M. tuberculosis* in CFA per mouse on Day 0 and received a booster with 0.75 mg SCH and 0.035 mg *M. tuberculosis* in CFA per mouse on Day 7. Dotted red lines indicate Day 49, before which the disease course was relapsing-remitting with two approximately synchronous waves of disease in most mice. After Day 49, some mice experienced a second relapse, but the disease was progressive in most mice. A relapse was defined as a period during which clinical scores rose at least 1 point above the trough of the preceding remission, with a peak sustained for at least two days, followed by a trough at least 0.5 clinical score below the peak lasting at least two days.

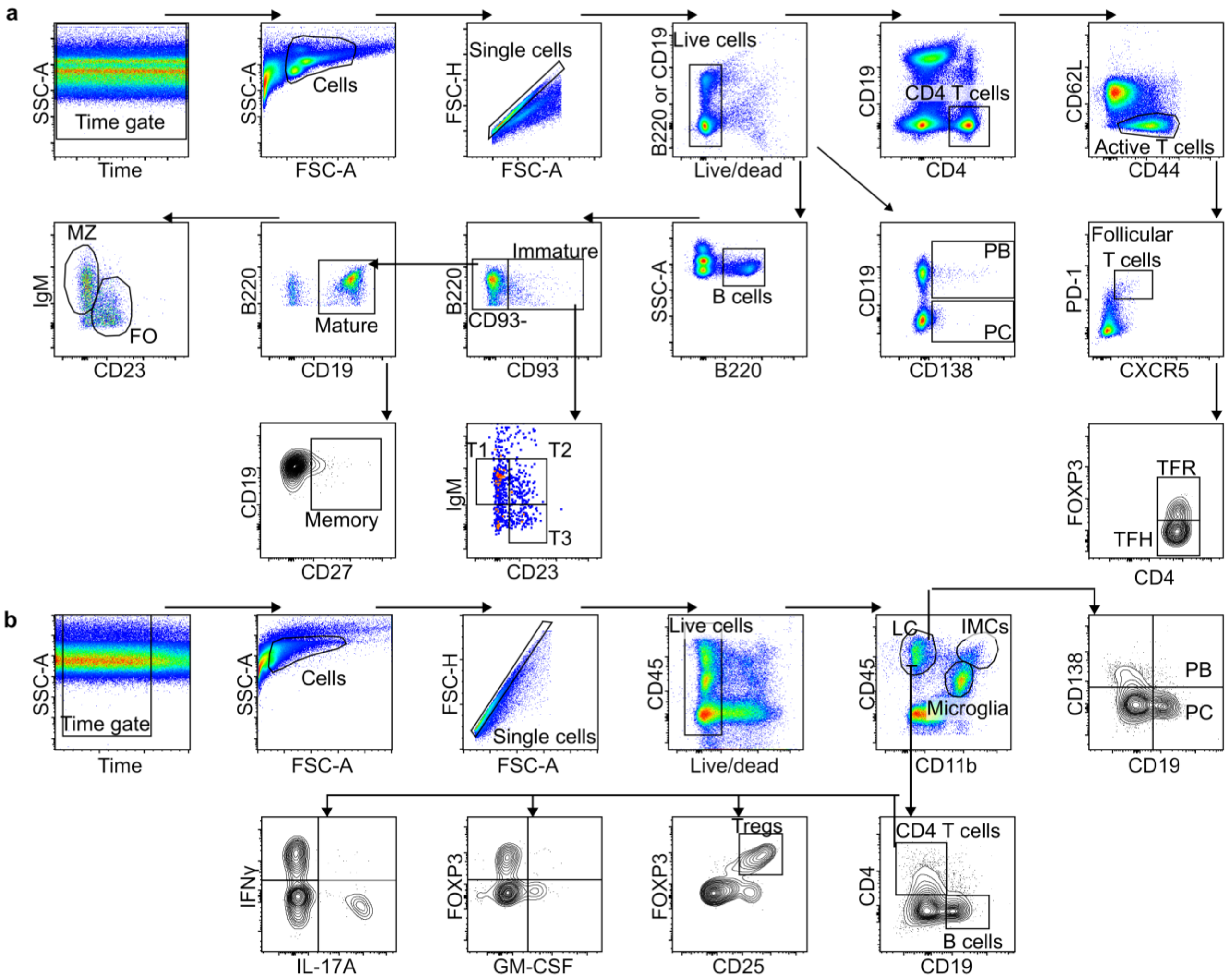

Figure S2 Gating strategies for flow cytometric analysis. Illustrative dot and contour plots demonstrating gating strategies used for flow cytometric analysis. **a** Strategy used to gate cell populations from mouse splenocytes. **b** Strategy used to gate on cells from CNS (brain plus spinal cord) or spinal cord. FO=follicular, IMCs=infiltrating myeloid cells, LC=lymphocytes, MZ=marginal zone, PB=plasmablasts, PC=plasma cells, TFH=T follicular helper, TFR=T follicular regulatory, Tregs=regulatory T cells.

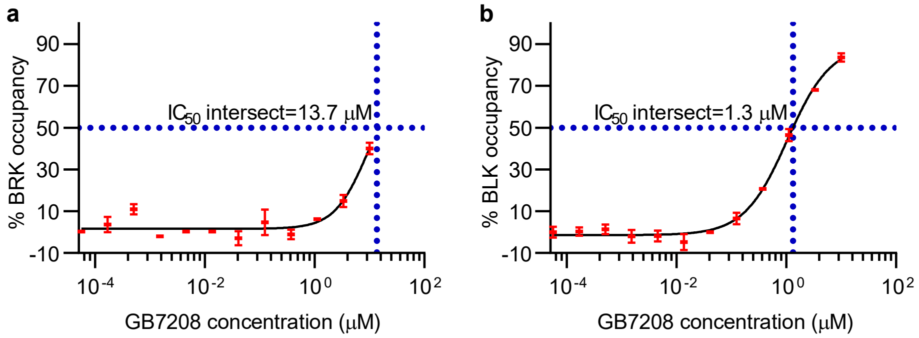

Figure S3 GB7208 inhibits BRK/PTK6 and BLK with IC_{50}s of 13.7 and 1.3 µM, respectively. Dose-response curves for percent BRK (**a**) and BLK (**b**) occupancy from triplicate values at each concentration of GB7208 using a nonlinear, 4-parameter, variable slope curve-fitting function. Data represent the mean±SD of 3 measurements performed in parallel.

SUPPLEMENTARY TABLES

Table S1 Antibodies used for flow cytometric analysis

Target	Clone	Conjugated fluorophore	Host	Company	Catalog #	RRID
Arg1	Met1Lys322	APC	Sheep	R&D	IC5868A	AB_2810265
B220/CD45R	RA3-6B2	Alexa Fluor 700	Rat	BioLegend	103232	AB_493717
CD11b	M1/70	Brilliant Violet 510	Rat	BioLegend	101263	AB_2629529
CD11b	M1/70	PerCP-Cy5.5	Rat	BioLegend	101228	AB_893232
CD138	281-2	PE-Cy7	Rat	BioLegend	142514	AB_2562198
CD19	6D5	PE-Cy5	Rat	BioLegend	115510	AB_313645
CD19	6D5	Brilliant Violet 711	Rat	BioLegend	115555	AB_2565970
CD19	6D5	Brilliant Violet 785	Rat	BioLegend	115543	AB_11218994
CD206	C068C2	Alexa Fluor 700	Rat	BioLegend	141733	AB_2629636
CD23	B3B4	APC	Rat	BioLegend	101620	AB_2563439
CD25	PC61	PE-Cy5	Rat	BioLegend	102010	AB_312859
CD27	LG.3A10	PE-Cy7	Hamster	BioLegend	124216	AB_10639726
CD4	RM4-5	Alexa Fluor 700	Rat	BioLegend	100536	AB_493701
CD4	GK1.5	Brilliant Violet 650	Rat	BioLegend	100469	AB_2783035
CD4	H129.19	PE-Cy5	Rat	BioLegend	130312	AB_2075572
CD44	IM7	PerCP-Cy5.5	Rat	BioLegend	103032	AB_2076204
CD45	30-F11	Brilliant Violet 605	Rat	BioLegend	103151	AB_2565884
CD45	30-F11	PE-Cy5	Rat	BioLegend	103110	AB_312975
CD62L	MEL-14	PE-Cy7	Rat	BioLegend	104417	AB_313102
CD86	GL-1	PE/Dazzle 594	Rat	BioLegend	105042	AB_2566409
CD93	AA4.1	PE	Rat	BioLegend	136503	AB_1967094
CXCR5 (CD185)	L138DF	PE	Rat	BioLegend	145504	AB_2561968
FOXP3	FJK-16s	FITC	Rat	eBioscience	11-5773-80	AB_465242
FOXP3	FJK-16s	APC	Rat	eBioscience	17-5773-82	AB_469457
GM-CSF	MP1-22E9	PE	Rat	BioLegend	505406	AB_315382
IFNγ	XMG1.2	Alexa Fluor 700	Rat	BioLegend	505824	AB_2561300
IgM	RMM-1	PE/Dazzle 594	Rat	BioLegend	406529	AB_2566585
IgM	RMM-1	Brilliant Violet 711	Rat	BioLegend	406539	AB_2814386
IL-10	JES5-16E3	FITC	Rat	BioLegend	505006	AB_315360
IL-17A	TC11-18H10.1	PE/Dazzle 594	Rat	BD Biosciences	506938	AB_2564321
iNOS (Nos2)	W16030C	PE	Rat	BioLegend	696806	AB_2876745
PD-1 (CD279)	29F.1A12	APC	Rat	BioLegend	135210	AB_2159183
Pro-IL-1β	NJTEN3	PE-Cy7	Rat	eBioscience	25-7114-82	AB_2573526
TNF	MP6-XT22	PE-Cy7	Rat	BioLegend	506324	AB_2256076

All listed antibodies are reactive against mouse antigens. RRID=Research Resource Identifier (https://scicrunch.org/resources).

Table S2 Splenocyte populations after prophylactic and semi-therapeutic ibrutinib treatment in EAE Biozzi mice

Cell population	Vehicle (n=7-8)	Ibrutinib Day 0 (n=8)	Post-test p value	Ibrutinib Day 9 (n=8)	Post-test p value	Test statistic$_{(d.f.)}$	Overall p value
**% of live cells**							
CD4 T cells	22.6±5.0	27.8±4.3	0.065	28.9±4.6	**0.025**	$F_{(2,21)}$=4.18	0.030
B cells	7.3±1.3	6.2±1.0	0.209	5.8±1.5	0.074	$F_{(2,21)}$=2.55	0.102
Plasmablasts	0.58±0.15	0.73±0.09	**0.026**	0.64±0.07	0.470	$F_{(2,21)}$=3.66	0.043
Plasma cells	1.08±0.33	0.84±0.34	0.430	0.94±0.54	0.745	$F_{(2,21)}$=0.651	0.531
**% of B cells**							
Immature	12.2±8.6	24.2±7.4	**0.007**	21.2±6.0	**0.045**	$F_{(2,21)}$=5.67	0.011
Mature	72.5±8.4	56.7±7.0	**<0.001**	60.1±5.4	**0.004**	$F_{(2,21)}$=11.1	<0.001
**% of immature B cells**							
T1	42.1±10.5	58.5±7.8	**0.002**	56.0±6.9	**0.007**	$F_{(2,21)}$=8.54	0.002
T2	11.2±3.2	14.1±1.9	**0.046**	11.8±1.9	0.840	$F_{(2,21)}$=3.23	0.060
T3	10.2±4.0	6.6±2.1	**0.031**	6.3±1.5	**0.022**	$F_{(2,21)}$=4.81	0.019
**% of mature B cells**							
MZ	47.9±3.8	34.7±6.2	**<0.001**	37.0±3.3	**<0.001**	$F_{(2,21)}$=18.3	<0.001
FO	39.0±3.9	53.8±5.4	**<0.001**	51.0±3.5	**<0.001**	$F_{(2,21)}$=26.7	<0.001
Memory	4.2±0.6	4.8±1.1	0.415	4.5±1.0	0.787	$F_{(2,21)}$=0.677	0.519
**# of cells (x10^5)**							
Live cells	599.5±63.4	742.6±160.8	0.085	741.4±140.7	0.087	$F_{(2,20)}$=2.88	0.080
CD4 T cells	142.8±38.0	207.2±58.2	**0.046**	214.6±54.8	**0.026**	$F_{(2,21)}$=4.28	0.028
B cells	44.5±7.6	46.2±11.3	0.937	43.4±12.8	0.971	$F_{(2,20)}$=0.133	0.877
Plasmablasts	3.6±0.7	5.4±1.5	**0.010**	4.7±0.9	0.129	$F_{(2,20)}$=4.92	0.018
Plasma cells	6.4±1.9	6.2±2.8	0.988	6.8±3.8	0.934	$F_{(2,20)}$=0.105	0.901
Immature B cells	4.7±3.3	11.1±4.4	**0.012**	9.5±4.4	0.060	$F_{(2,20)}$=4.92	0.018
Mature B cells	33.3±6.2	26.4±7.9	0.163	26.2±8.4	0.150	$F_{(2,20)}$=2.06	0.153
T1 B cells	2.0±1.7	6.6±2.9	**0.005**	5.4±2.8	**0.032**	$F_{(2,20)}$=6.41	0.007
T2 B cells	0.5±0.3	1.5±0.5	**<0.001**	1.1±0.5	**0.030**	$F_{(2,20)}$=10.5	<0.001
T3 B cells	0.5±0.3	0.7±0.4	0.281	0.6±0.3	0.772	$F_{(2,20)}$=1.03	0.375
MZ B cells	15.9±3.3	9.4±3.7	**0.003**	9.7±3.1	**0.004**	$F_{(2,20)}$=8.61	0.002
FO B cells	13.1±2.9	14.0±3.9	0.861	13.4±4.6	0.982	$F_{(2,20)}$=0.107	0.899
Memory B cells	1.4±0.3	1.3±0.4	0.453	1.1±0.2	0.132	$F_{(2,20)}$=1.76	0.198

Analyses were performed on n=7-8 representative mice/group from the experiment shown in Fig. 2a. Values for CD4 T cells, plasmablasts, and plasma cells are from cells stimulated as described in the Methods section. MZ=marginal zone, FO=follicular. Values are shown as mean±SD. Significance for proportions (%) and numbers (#) of cells was tested using one-way ANOVAs followed by Dunnett's multiple comparisons tests. Bold p values indicate a significant difference vs. the Vehicle group.

Table S3 Histological outcomes in spinal cords of prophylactic and semi-therapeutic ibrutinib treatment in EAE Biozzi mice

**Spinal cord region**	**Vehicle (n=11)**	**Ibrutinib Day 0 (n=13)**	**Post-test p value**	**Ibrutinib Day 9 (n=12)**	**Post-test p value**	**Test statistic$_{(d.f.)}$**	**Overall p value**
**Demyelination score**							
Cervical	1.3±1.3	0.1±0.3	**0.003**	0.2±0.4	**0.014**	$H_{(2)}$=11.5	0.003
Thoracic	1.7±1.6	0.2±0.4	**0.007**	0.3±0.5	**0.027**	$H_{(2)}$=9.66	0.008
Lumbar	2.2±1.6	0.5±0.7	**0.009**	0.7±1.2	**0.021**	$H_{(2)}$=9.59	0.008
Average	1.7±1.3	0.2±0.4	**0.001**	0.4±0.5	**0.014**	$H_{(2)}$=12.8	0.002
**Inflammation (foci/section)**							
Cervical	2.9±3.3	0.4±1.1	**0.007**	0.3±0.6	**0.005**	$F_{(2,33)}$=6.71	0.004
Thoracic	3.5±3.7	0.4±0.9	**0.004**	0.8±1.4	**0.012**	$F_{(2,33)}$=6.52	0.004
Lumbar	5.0±5.1	1.7±3.0	0.080	2.5±3.3	0.222	$F_{(2,33)}$=2.31	0.115
Average	3.8±3.7	0.8±1.4	**0.009**	1.2±1.5	**0.023**	$F_{(2,33)}$=5.35	0.010
**Apoptosis (nuclei/section)**							
Cervical	0.1±0.3	0.0±0.0	0.314	0.0±0.0	0.326	$F_{(2,33)}$=1.15	0.330
Thoracic	0.6±0.9	0.1±0.3	**0.029**	0.0±0.0	**0.014**	$F_{(2,33)}$=4.80	0.015
Lumbar	0.3±0.5	0.0±0.0	0.073	0.1±0.3	0.274	$F_{(2,33)}$=2.43	0.103
Average	0.3±0.4	0.0±0.1	**0.005**	0.0±0.1	**0.007**	$F_{(2,33)}$=6.63	0.004

All surviving mice from the experiment shown in Fig. 2a were used in this analysis. For each stain, 1 section per mouse containing cervical, thoracic, and lumbar regions of spinal cord (3 regions per slide) was prepared and analyzed. "Average" was calculated by taking the mean value of the 3 analyzed regions. Values are shown as mean±SD. Significance for demyelination scores was tested using a Kruskal-Wallis test followed by Dunn's multiple comparisons test. Significance for numbers of inflammatory foci and numbers of apoptotic cells was tested using one-way ANOVAs followed by Dunnett's multiple comparisons tests. Bold p values indicate a significant difference vs. the Vehicle group.

Table S4 Histological outcomes in spinal cords of late therapeutic ibrutinib treatment in EAE Biozzi mice

Spinal cord region	Vehicle (n=12)	Ibrutinib Day 49 (n=12)	Test statistic$_{(d.f.)}$	p value
**Demyelination score**				
Cervical	1.6±1.2	0.5±0.7	U=35.5	**0.034**
Thoracic	1.7±0.7	1.0±0.6	U=36.0	**0.035**
Lumbar	1.6±0.9	0.7±0.7	U=31.5	**0.017**
Average	1.6±0.6	0.7±0.4	U=18.0	**<0.001**
**Inflammation (foci/section)**				
Cervical	2.5±2.8	0.8±0.7	$t_{(22)}$=1.99	0.059
Thoracic	1.1±1.4	0.8±0.8	$t_{(22)}$=0.537	0.596
Lumbar	2.1±1.0	1.3±1.0	$t_{(22)}$=1.85	0.077
Average	1.9±1.3	1.0±0.7	$t_{(22)}$=2.16	**0.042**
**Apoptosis (nuclei/section)**				
Cervical	0.2±0.4	0.2±0.4	$t_{(22)}$=0.00	>0.999
Thoracic	0.2±0.6	0.2±0.4	$t_{(22)}$=0.00	>0.999
Lumbar	0.1±0.3	0.0±0.0	$t_{(22)}$=1.00	0.328
Average	0.1±0.3	0.1±0.2	$t_{(22)}$=0.235	0.816

Analyses were performed on n=12 representative mice/group from the experiment shown in Fig. 4a. For each stain, 1 section per mouse containing cervical, thoracic, and lumbar regions of spinal cord (3 regions per slide) was prepared and analyzed. "Average" was calculated by taking the mean value of the 3 analyzed regions. Values are shown as mean±SD. Significance for demyelination scores was tested using two-tailed Mann-Whitney tests. Significance for numbers of inflammatory foci and numbers of apoptotic cells was tested using two-tailed unpaired t tests. Bold p values indicate a significant difference vs. the Vehicle group.

Table S5 Splenocyte populations after late therapeutic ibrutinib treatment in EAE Biozzi mice

Cell population	Vehicle (n=6-9)	Ibrutinib Day 49 (n=6-10)	Test statistic$_{(d.f.)}$	p value
**% of live cells**				
CD4 T cells	30.1±2.7	28.3±2.1	$t_{(17)}$=1.56	0.138
B cells	29.2±4.0	25.7±3.3	$t_{(10)}$=1.64	0.131
Plasmablasts	1.4±0.5	1.2±0.2	$t_{(10)}$=0.818	0.433
Plasma cells	3.6±0.8	4.6±0.8	$t_{(10)}$=2.22	0.050
**% of CD4 T cells**				
TFH	0.19±0.09	0.09±0.03	$t_{(17)}$=3.48	**0.003**
TFR	0.12±0.03	0.08±0.03	$t_{(17)}$=3.14	**0.006**
**% of B cells**				
Immature	4.9±2.1	11.7±4.7	$t_{(10)}$=3.26	**0.009**
Mature	93.1±2.4	85.0±5.0	$t_{(10)}$=3.63	**0.005**
**% of immature B cells**				
T1	54.0±7.8	63.3±5.6	$t_{(10)}$=2.37	**0.039**
T2	28.2±8.6	29.4±5.1	$t_{(10)}$=0.303	0.768
T3	8.9±3.6	3.6±1.8	$t_{(10)}$=3.22	**0.009**
**% of mature B cells**				
MZ	46.7±11.2	29.8±4.3	$t_{(10)}$=3.45	**0.006**
FO	50.2±11.4	67.6±4.4	$t_{(10)}$=3.49	**0.006**
Memory	3.9±1.8	11.2±4.3	$t_{(10)}$=3.82	**0.003**
**# of cells (x10^5)**				
Live cells	727.1±245.4	446.7±95.5	$t_{(10)}$=2.61	**0.026**
CD4 T cells	256.3±53.4	207.0±38.5	$t_{(17)}$=2.33	**0.033**
B cells	215.6±76.7	116.0±32.5	$t_{(10)}$=2.93	**0.015**
Plasmablasts	9.6±4.0	5.6±2.2	$t_{(10)}$=2.17	0.055
Plasma cells	27.0±10.4	20.8±6.1	$t_{(10)}$=1.26	0.236
TFH cells	0.51±0.34	0.17±0.06	$t_{(17)}$=3.13	**0.006**
TFR cells	0.30±0.10	0.16±0.05	$t_{(17)}$=3.35	**0.004**
Immature B cells	11.5±7.1	13.5±6.0	$t_{(10)}$=0.520	0.615
Mature B cells	199.8±69.4	98.8±29.2	$t_{(10)}$=3.28	**0.008**
T1 B cells	5.8±3.2	8.6±4.1	$t_{(10)}$=1.34	0.211
T2 B cells	3.6±2.6	4.0±2.1	$t_{(10)}$=0.281	0.784
T3 B cells	1.2±1.1	0.4±0.2	$t_{(10)}$=1.67	0.126
MZ B cells	89.7±34.6	28.8±7.2	$t_{(10)}$=4.23	**0.002**
FO B cells	104.0±43.3	67.4±22.0	$t_{(10)}$=1.85	0.095
Memory B cells	7.5±4.3	10.9±5.2	$t_{(10)}$=1.25	0.239

For all CD4 T cell populations, analyses were performed on all surviving mice (n=9-10/group) from the experiment shown in Fig. 4c. For all other populations, data are representative of two independent experiments and are from n=6 representative mice/group from the experiment shown in Fig. 4a. Values for CD4 T cells, TFH cells, TFR cells, plasmablasts, and plasma cells are from cells stimulated as described in the Methods section. MZ=marginal zone, FO=follicular, TFH=T follicular helper, TFR=T follicular regulatory. Values are shown as mean±SD. Significance for proportions (%) and numbers (#) of cells was tested using two-tailed unpaired t tests. Bold p values indicate a significant difference vs. the Vehicle group.

Table S6 Lymphocyte populations in the CNS after late therapeutic ibrutinib treatment in EAE Biozzi mice

Cell population	Vehicle (n=6)	Ibrutinib Day 49 (n=6)	Test $statistic_{(d.f.)}$	p value
**% of live cells**				
Lymphocytes	20.8±8.9	23.0±7.9	$t_{(10)}$=0.462	0.654
CD4 T cells	1.8±0.6	3.1±2.1	$t_{(10)}$=1.78	0.176
B cells	4.0±2.5	2.7±1.3	$t_{(10)}$=1.07	0.308
Plasmablasts	0.15±0.04	0.19±0.07	$t_{(10)}$=1.06	0.313
Plasma cells	2.1±0.8	2.8±1.0	$t_{(10)}$=1.47	0.173
**% of CD4 T cells**				
GM-CSF^{+}	3.4±1.4	3.4±1.0	$t_{(10)}$=0.120	0.907
$IFN\gamma^{+}IL17\text{-}A^{-}$	15.0±9.2	9.3±2.0	$t_{(10)}$=1.46	0.176
$IFN\gamma^{+}IL17\text{-}A^{+}$	1.0±0.5	0.9±0.3	$t_{(10)}$=0.641	0.536
$IFN\gamma^{-}IL17\text{-}A^{+}$	12.3±1.7	16.1±5.1	$t_{(10)}$=1.74	0.112
Tregs	18.9±2.9	21.3±4.5	$t_{(10)}$=1.10	0.296
**# of cells ($x10^3$)**				
Live cells	454.7±109.7	511.3±98.1	$t_{(10)}$=0.942	0.368
Lymphocytes	97.8±60.4	117.6±45.0	$t_{(10)}$=0.645	0.533
Plasmablasts	0.69±0.22	0.95±0.33	$t_{(10)}$=1.61	0.138
Plasma cells	9.3±3.8	14.4±5.5	$t_{(10)}$=1.84	0.095
CD4 T cells	8.0±3.7	14.7±9.5	$t_{(10)}$=1.52	0.136
B cells	19.1±16.3	14.3±7.0	$t_{(10)}$=0.665	0.521

CNS (brain and spinal cord combined) suspensions were generated and stimulated as described in the Methods section from n=6 representative mice/group from the experiment shown in Fig. 4a. Tregs=regulatory T cells. Values are shown as mean±SD. Significance for proportions (%) and numbers (#) of cells was tested using two-tailed unpaired t tests. Data are representative of two independent experiments.

Table S7 Activation markers in spinal cord myeloid cells after late therapeutic ibrutinib treatment in EAE Biozzi mice

Readout	Vehicle (n=11)	Ibrutinib Day 49 (n=12)	Post-test p value	Naïve (n=6)	Post-test p value	Test statistic$_{(d.f.)}$	Overall p value
# live cells (x10^3)	14.7±7.0	10.4±4.3	0.114	5.2±3.3	**0.003**	$F_{(2,26)}$=6.24	0.006
**Infiltrating myeloid cells (IMCs)**							
% of live	7.7±3.9	6.6±2.8	0.580	2.7±0.9	**0.006**	$F_{(2,26)}$=5.54	0.010
# (x10^3)	1.20±0.82	0.63±0.30	**0.037**	0.14±0.12	**0.002**	$F_{(2,26)}$=7.69	0.002
CD86 (MFI)	268±100	197±61	0.060	102±27	**<0.001**	$F_{(2,26)}$=9.67	0.001
Pro-IL-1β (MFI)	2079±510	1498±412	**0.008**	1354±373	**0.007**	$F_{(2,26)}$=7.00	0.004
TNF (MFI)	1523±243	1459±206	0.768	1025±318	**<0.001**	$F_{(2,26)}$=8.74	0.001
iNOS (MFI)	1032±264	874±176	0.165	744±189	**0.027**	$F_{(2,26)}$=3.69	0.039
Arg1 (MFI)	615±247	1009±240	**0.003**	1120±338	**0.002**	$F_{(2,26)}$=9.43	<0.001
CD206 (MFI)	1926±526	1020±590	**0.001**	783±539	**<0.001**	$F_{(2,26)}$=11.1	<0.001
IL-10 (MFI)	110±24	92±17	0.095	63±24	**<0.001**	$F_{(2,26)}$=9.41	<0.001
**Microglia**							
% of live	44.2±14.0	51.9±15.0	0.292	85.9±1.4	**<0.001**	$F_{(2,26)}$=20.8	<0.001
# (x10^3)	6.8±4.5	5.4±2.7	0.548	4.4±2.8	0.341	$F_{(2,26)}$=0.952	0.399
CD86 (MFI)	288±58	251±15	**0.048**	152±6	**<0.001**	$F_{(2,26)}$=25.9	<0.001
Pro-IL-1β (MFI)	891±77	782±66	**<0.001**	750±29	**<0.001**	$F_{(2,26)}$=12.0	<0.001
TNF (MFI)	548±49	528±61	0.665	603±81	0.163	$F_{(2,26)}$=2.97	0.069
iNOS (MFI)	515±33	489±40	0.143	434±15	**<0.001**	$F_{(2,26)}$=11.2	<0.001
Arg1 (MFI)	73±22	103±23	**0.004**	62±6	0.441	$F_{(2,26)}$=10.0	<0.001
CD206 (MFI)	1337±107	1200±132	**0.012**	996±45	**<0.001**	$F_{(2,26)}$=18.6	<0.001
IL-10 (MFI)	94±9	100±6	0.075	78±4	**<0.001**	$F_{(2,26)}$=21.0	<0.001

Age-matched naïve mice and all surviving mice from the experiment shown in Fig. 4e were used in this analysis. Values for TNF and IL-10 are from cells stimulated as described in the Methods section. MFI=median fluorescence intensity. Values are shown as mean±SD. For each readout, groups were compared to the Vehicle group using a one-way ANOVA followed by Dunnett's multiple comparisons test. Bold p values indicate a significant difference vs. the Vehicle group.

Table S8 Spinal cord Iba1+ and SMI32+ tissue area after late therapeutic ibrutinib treatment in EAE Biozzi mice

Spinal cord region	Vehicle (n=12)	Ibrutinib Day 49 (n=12)	Test $statistic_{(d.f.)}$	p value
**% Iba1+ of spinal cord area**				
Cervical	6.4±3.5	3.9±1.5	$t_{(22)}$=2.25	**0.035**
Thoracic	6.4±2.8	4.0±1.8	$t_{(21)}$=2.48	**0.022**
Lumbar	7.1±2.6	5.1±2.2	$t_{(21)}$=2.01	0.057
Average	6.6±2.2	4.3±1.6	$t_{(22)}$=2.95	**0.007**
**%SMI32+ of white matter area**				
Cervical	0.69±0.28	0.44±0.22	$t_{(22)}$=2.54	**0.019**
Thoracic	1.13±0.55	1.17±0.48	$t_{(22)}$=0.182	0.858
Lumbar	0.94±0.29	0.61±0.22	$t_{(21)}$=3.18	**0.005**
Average	0.93±0.18	0.74±0.23	$t_{(22)}$=2.25	**0.035**

Analyses were performed on n=12 representative mice/group from the experiment shown in Fig. 4a. For each stain, 1 section per mouse containing cervical, thoracic, and lumbar regions of spinal cord (3 regions per slide) was prepared and analyzed. "Average" was calculated by taking the mean value of the 3 analyzed regions. Values are shown as mean±SD. Significance for proportion of total tissue area positive for each marker was tested using two-tailed unpaired t tests. Bold p values indicate a significant difference vs. the Vehicle group.

Table S9 In vitro measures of BTK inhibitor potency

Readout	Ibrutinib	n	GB7208	n
BTK biochemical IC50 (nM)	1.4 ± 0.6	89	2.4 ± 1.3	9
HWB k_{inact}/K_i (10^{-4} nM^{-1} min^{-1})	0.7 ± 0.5	4	3.2 ± 1.7	7
Ramos calcium flux IC50 (nM)	14.6 ± 4.3	6	7.4 ± 2.6	7
Human B cell proliferation IC50 (nM)	0.4 ± 0.1	2	0.5 ± 0.1	3

Values are shown as mean±SD. HWB=human whole blood.
